# BMX, a specific HDAC8 inhibitor, with TMZ for advanced CRC therapy: a novel synergic effect to elicit p53-, β-catenin- and MGMT-dependent apoptotic cell death

**DOI:** 10.1186/s12964-022-01007-x

**Published:** 2022-12-27

**Authors:** Huey-Jiun Ko, Shean-Jaw Chiou, Cheng-Yu Tsai, Joon-Khim Loh, Xin-Yi Lin, Thu-Ha Tran, Chia-Chung Hou, Tai-Shan Cheng, Jin-Mei Lai, Peter Mu-Hsin Chang, Feng-Sheng Wang, Chun-Li Su, Chi-Ying F. Huang, Yi-Ren Hong

**Affiliations:** 1 Graduate Institutes of Medicine, College of Medicine, Kaohsiung Medical University, Kaohsiung, 80708 Taiwan; 2 Department of Biochemistry, College of Medicine, Kaohsiung Medical University, Kaohsiung, 80708 Taiwan; 3 Post Baccalaureate Medicine, Kaohsiung Medical University, Kaohsiung, 80708 Taiwan; 4 Department of Neurosurgery, Kaohsiung Medical University Hospital, Kaohsiung, 80708 Taiwan; 5 Taiwan International Graduate Program in Molecular Medicine, National Yang Ming Chiao Tung University and Academia Sinica, Taipei, 112 Taiwan; 6 Institute of Biopharmaceutical Sciences, National Yang Ming Chiao Tung University, Taipei, 11211 Taiwan; 7 New Drug Research & Development Center, NatureWise Biotech & Medicals Corporation, Taipei, 112 Taiwan; 8 Department of Life Science, Fu-Jen Catholic University, New Taipei City, 24205 Taiwan; 9 Department of Oncology, Taipei Veterans General Hospital, Taipei, 11217 Taiwan; 10 Faculty of Medicine, National Yang Ming Chiao Tung University, Taipei, 11211 Taiwan; 11 Department of Chemical Engineering, National Chung Cheng University, Chiayi, 62102 Taiwan; 12 Graduate Program of Nutrition Science, School of Life Science, National Taiwan Normal University, Taipei, 11677 Taiwan; 13 Department of Biotechnology and Laboratory Science in Medicine, National Yang Ming Chiao Tung University, Taipei, 11211 Taiwan; 14 Department of Biological Sciences, National Sun Yat-Sen University, Kaohsiung, 804 Taiwan; 15 Department of Medical Research, Kaohsiung Medical University Hospital, Kaohsiung, 80708 Taiwan; 16 Center for Cancer Research, Kaohsiung Medical University, Kaohsiung, 80708 Taiwan; 17 Neuroscience Research Center, Kaohsiung Medical University, Kaohsiung, 80708 Taiwan

**Keywords:** HDAC8 inhibitor, CRC, TMZ, MGMT, β-catenin, p53

## Abstract

**Background:**

Despite advances in treatment, patients with refractory colorectal cancer (CRC) still have poor long-term survival, so there is a need for more effective therapeutic options.

**Methods:**

To evaluate the HDAC8 inhibition efficacy as a CRC treatment, we examined the effects of various HDAC8 inhibitors (HDAC8i), including BMX (NBM-T-L-BMX-OS01) in combination with temozolomide (TMZ) or other standard CRC drugs on p53 mutated HT29 cells, as well as wild-type p53 HCT116 and RKO cells.

**Results:**

We showed that HDAC8i with TMZ cotreatment resulted in HT29 arrest in the S and G2/M phase, whereas HCT116 and RKO arrest in the G0/G1 phase was accompanied by high sub-G1. Subsequently, this combination approach upregulated p53-mediated MGMT inhibition, leading to apoptosis. Furthermore, we observed the cotreatment also enabled triggering of cell senescence and decreased expression of stem cell biomarkers. Mechanistically, we found down-expression levels of β-catenin, cyclin D1 and c-Myc via GSK3β/β-catenin signaling. Intriguingly, autophagy also contributes to cell death under the opposite status of β-catenin/p62 axis, suggesting that there exists a negative feedback regulation between Wnt/β-catenin and autophagy. Consistently, the Gene Set Enrichment Analysis (GSEA) indicated both apoptotic and autophagy biomarkers in HT29 and RKO were upregulated after treating with BMX.

**Conclusions:**

BMX may act as a HDAC8 eraser and in combination with reframed-TMZ generates a remarkable synergic effect, providing a novel therapeutic target for various CRCs.

**Video Abstract**

**Supplementary Information:**

The online version contains supplementary material available at 10.1186/s12964-022-01007-x.

## Introduction

Colon cancer or colorectal cancer (CRC) is one of the most prevalent kinds of malignancy tumors and the third leading cause of cancer mortality globally. Although the standard treatment for colon cancer or CRC has been well studied and established, the mortality rate remains high and a number of related clinical issues have yet to be solved. Disease symptoms are less apparent as patients are frequently diagnosed with advanced cancer at the initial evaluation, and the subsequent five-year survival rate is around 10% [1, 2]. CRC tumors can be classified into four biologically distinct consensus molecular subtypes (CMS1-4) by transcriptomic features [3]. The standard treatments of CRC are surgery, radiation and/or chemotherapy, in which Oxaliplatin (Oxp) and its prodrug capecitabine are widely used in clinical practice [4, 5]. Unfortunately, recurrence under this kind of DNA crosslink agent treatment is still common within the first few years even after completing the whole cycle [6].

It is well known that the predominant mechanism of resistance to temozolomide (TMZ) is the expression of O6-methylguanine-DNA-methyltransferase (MGMT), a DNA repair enzyme that removes the O6-methyl-guanine DNA adducts in a one-step suicide reaction. A comparison of the MGMT protein level between newly diagnosed and recurrent CRC patients who had received TMZ treatment supports the notion that MGMT reduction may promote the efficacy of TMZ treatment [7–9]. In several clinical trials on metastatic CRC, an impressive clinical response to TMZ in patients with MGMT loss of expression was reported in 27–40% of CRC patients [10–14]. Clearly, TMZ alone does not provide a promising clinical benefit in CRC, and the role of TMZ alone in CRC is still controversial [15]. However, TMZ is an oral alkylating agent with a good safety profile, and TMZ methylates several sites within the DNA, such as 7-methylguanosine (m7G, 70%), which is the most abundant product, followed by 3-methyladenosine (m3A, 10–20%) and O6-methylguanosine (O6mG, 5%) [16]. During DNA replication, O6mG will mismatch to thymine (T) and trigger a double-strand break because of mismatch repair failure, unless MGMT removes the methyl adduct. Similar to O6mG, m7G triggers single-strand DNA breaks because of base excision repair failure, which can be rescued by Poly ADP-ribose Polymerase 1 (PARP-1) [12]. In addition to the DNA replication error by O6mG and m7G, TMZ also causes robust G2/M arrest, indicating the potential existence of other cytotoxicity mechanisms [17, 18]. Based on this notion, the synergistic effect of TMZ and other compounds is still a promising target.

BMX (NBM-T-L-BMX-OS01), a histone deacetylase 8 inhibitor (HDAC8i), shows significant anti-cell proliferation effects in CRC cells, human umbilical endothelial cells, lung cancer cells, and glioblastoma cells, and it also exhibits tumor suppression ability in an animal xenograft model [8, 19, 20]. These anti-cell proliferation and apoptosis activation effects result from HDAC8-modified non-histone proteins, such as p53, which elevate its protein and acetylation level after knocking down HDAC8 [21–23]. Recently, the results of our studies suggested that HDAC8i could overcome TMZ resistance in glioblastoma multiforme (GBM) by downregulating the β-catenin/c-Myc/SOX2 pathway and upregulating p53-mediated MGMT inhibition [8]. Therefore, the inhibition of HDAC8 as an epigenetic tool has emerged as an effective treatment strategy for cancer therapy [24].

In this study, we optimized the concentration of HDAC8i BMX with TMZ to treat CRC cell lines. Our results show a combination of BMX and TMZ triggers cell cycle arrest, senescence, autophagy, and apoptosis in CRC cells via upregulation of p53/p21/E2F3/Bax, which in turn was compromised by the crosstalk of the downregulating Wnt/β-catenin/cyclin D1/c-Myc /p62 pathways. We demonstrated the utility of a highly specific HDAC8 inhibitor BMX used in combination with TMZ to generate a synergic effect, which may provide a promising new therapeutic target for CRC patients.

## Materials and methods

### Cell lines and cell culture

Three CRC cell lines, HT29, HCT116, and RKO, were used in this study. The American Type Culture Collection (ATCC; Manassas, VA, USA) provided human CRC cell lines HT29 (ATCC HTB-38; mutant p53, p.R273H; APC frame shift, p. E1554fs; wild-type β-catenin), HCT116 (ATCC CCL-247; wild-type p53; wild-type APC; deletion β-catenin, p. S45del) and RKO (ATCC CRL-2577; wild-type p53; wild-type APC; wild-type β-catenin). The three CRC cell lines listed above were cultured in an adherent culture condition, maintained at 37℃ inside a cell incubator containing 5% CO_2_. Cells from the HCT-116 and HT-29 cell lines were cultured in McCoy’s 5A medium supplemented with 10% fetal bovine serum (FBS; Gibco; Thermo Fisher Scientific, Grand Island, NY, USA), 1% penicillin, and 1% streptomycin. RKO cells were cultured in MEM medium supplemented with 10% FBS, 1% penicillin, 1% streptomycin, and 1% sodium pyruvate. The cell cultures were passaged by trypsinization every three days. BMX, (E)-2-(4-Methoxybenzyloxy)-3-prenyl-4-methoxy-N-hydroxycinamide, was provided by Nature Wise Biotech & Medicals Corporation (Taipei, Taiwan).

### Cell proliferation assays

The cell proliferation of CRC cells was measured by CCK8 assay (Targetmol, Shanghai, China). Cells (4 × 10^3^) were seeded in 96-well plates and allowed to adhere overnight, followed by treatment with different doses of BMX (0–10 µM), VPA (4 mM), SAHA (2 µM) with or without TMZ (50 µM), Oxp (5 µM) and Dox (1 µM) for 24, 48, and 72 h. One-tenth of the medium volume of CCK-8 reagent was added to each well at the indicated time points. After 1–4 h of CCK-8 reagent treatment, the proliferation of cells was determined by microplate reader (Multiskan GO, Thermo Fisher Scientific, Waltham, MA, USA) at 450 nm. Experiments were repeated at least 3 times independently and the results are presented as bar diagrams along with the mean ± standard.

### Cell cycle analysis

Cells were treated with different doses of BMX (5 and 10 μM) in the presence or absence of TMZ (50 μM) for 48 h. Cells were fixed with methanol and then stained with PI working solution (PI, 10 µg/mL and RNase A, 20 mg/mL) for 15 min in the dark. Using a flow cytometer (Attune NxT flow cytometer, Thermo Fisher Scientific), the PI fluorescence of 10,000 individual nuclei was calculated. Attune software was used to evaluate the fractions of the cells based on the mean fluorescence intensity values.

### Apoptosis assay

Apoptosis was investigated by CF®488A Annexin V and PI double staining assay (Fremont, CA, USA). Cells (5 × 10^6^) were collected, washed with PBS and then suspended in 100 μL/tube of binding buffer (adding 5 μL of annexin V-FITC and 0.1 μL of PI). After incubating for 15–30 min in the dark, 400 μL of binding buffer was added to samples and immediately analyzed by flow cytometry.

### Quantitative real time RT-PCR

RNA was extracted from the cells (2 × 10^5^) using Tissue Total RNA Mini Kit (Geneaid, Taipei, Taiwan) following the manufacturer’s instructions. cDNA was synthesized using a High-Capacity cDNA Reverse Transcription Kit (Applied Biosystems). qPCR reactions were performed using a 7500 Real-time PCR System (Applied Biosystems) with a Power SYBR Green PCR Master Mix (Applied Biosystems), according to the manufacturer’s recommendations, with 18 s as the inner reference. The cycle threshold (Ct) values were calculated using the StepOnePlus (Applied Biosystems) software. The relative expression of each mRNA was calculated using the 2 − (ΔCt) method. The primer sequences for HDAC8 were as follows: HDAC8 forward 5’-GCGTGATTTCCAGCACATAA-3’; HDAC8 reverse 5’-ATACTTGACCGGGGTCATCC-3’. follows: MGMT forward 5’-ACCGTTTGCGACTTGGTACT-3’; MGMT reverse 5’-TGCTCACAACCAGACAGCTC-3’. 18 s forward 5’-TCAAGTGCAGTGCAACAACTC-3’; 18 s reverse 5’-AGAGGACAGGGTGGAGTAATCA-3’.

### Clonogenic assay

Cells (2 × 10^3^/well) were seeded in 6-well plates and treated with BMX, VPA, and SAHA in the presence or absence of TMZ. The drugs and medium were changed every 2–3 days after outgrowth of cells. After 14 days, cells were washed and fixed with 4% paraformaldehyde for 30 min, followed by staining with 0.1% crystal violet for 30 min. The crystal violet-stained cells were solubilized in DMSO and the intensity was quantified by the absorbance at 570 nm. The results are expressed as the average colony ± SD from three independent experiments.

### Senescence-associated (SA) β-galactosidase (SA-β-gal) analysis

SA expression of β-gal activity was done with a Senescence Detection kit (CS0030-1KT; Sigma-Aldrich; Merck Millipore, Darmstadt, Germany). Briefly, cells washed with PBS and fixed using the fixative solution for half an hour at room temperature, followed by incubation at 37 °C overnight with the SA-β-gal staining solution. SA-β-gal activity was examined by X-gal (5-bromo-4-chloro-3-3indolyl β-D-galactoside) staining at pH 6.0. The blue-stained senescent cells were photographed. Randomly selected fields (n = 3) were analyzed by light microscopy to quantify the percentage of senescent cells.

### Western blot analysis

Cells were seeded at a density of 1 × 10^6^/10 cm dish. Following treatment with BMX (0, 5, and 10 μM), and suberoylanilide hydroxamic acid (SAHA), Valproic acid (VPA) or PCI-34051 in the presence or absence of TMZ (50 μM) or Oxp (5 μM) for 48 h, the lysates were analyzed by Western blotting as described previously [8]. The specific primary antibodies against acetyl-histone H3 (Lys9/Lys14), acetyl-histone H4 (Lys8), p53, acetyl-p53 (Lys382), phospho-p53 (Ser15), p21, p16, MGMT, phosphor-γ-H2AX (Ser139), E2F1, E2F3, cleaved caspase 3, cleaved caspase 8, cleaved caspase 7, cleaved caspase 9, PARP, Bax, Bcl-2, Bid, Bim, Bak, Puma, β-catenin, phospho-β-catenin (Ser/33/37/41), GSK3β, phospho-GSK3β (Ser 9), c-Myc, Cyclin D1, p62, LC3I/II, CD133, CD44, SOX-2, and HDAC8 were used for detection, and GAPDH, α-tubulin or β-actin was used as the internal control. After incubation with the primary antibodies, followed by incubation with horseradish peroxidase (HRP)-conjugated secondary antibodies, the intensities were developed using a chemiluminescent solution (Pierce, Rockford, US) and detected using a gel imager CCD camera (MultiGel-21, Topbio, Taipei, Taiwan). All antibodies and their dilutions are shown in Additional file 1: Table S1.

### Next-generation sequencing (NGS)

HT29 and RKO cells were treated with BMX at a concentration of 10 µM for 6 h. Total RNA from BMX treatment and control samples in HT29 and RKO cells was extracted using an RNeasy Mini kit (Qiagen). The mRNA expression level of each sample was detected using next-generation sequencing-RNAseq (Biotools Microbiome Research Center Inc.). The raw read counts were normalized using “Trimmed Mean of M-values” via edgeR (v3.8.1), and biologically unduplicated differentially expressed gene (DEGs) analysis was performed with the DEGseq package (v1.40.0) using the MARS (MA-plot-based method with Random Sampling model) method.

### Gene set enrichment analysis (GSEA)

The NGS profiling results were analyzed using Gene Set Enrichment Analysis (GSEA) software, version 4.0.3. This analysis compares the ranking of most altered gene expression under the drug treatment with published gene sets of various pathways to determine the level of similarity. GSEA assigns an enrichment score based on the Kolmogorov–Smirnov statistic for each gene set and then normalizes the score based on their size. Based on the normalized enrichment score, a permutation-based false discovery rate is generated to indicate the significance of the enrichment score. The analysis was conducted using the C2 (canonical pathways) gene set collections from the MSigDB v.7.2. with 1000 permutations. In addition, to determine the pathways affected by BMX treatment in both HT29 and RKO cells, the downregulated genes from both cells were overlapped using Venny 2.1 and then entered into the ConsensusPathDB to obtain enriched pathway-based sets from the Reactome database with a cut-off q-value of < 0.001 and a selection of pathways containing more than 4 candidates.

### Combination index (CI) analysis

Drug toxicity was determined via CCK-8 assay for 24, 48, and 72 h in HT29, HCT116 and RKO cells. The CI was calculated via Compusyn software (www.combosyn.com; version 1.0; ComboSyn, Paramus, NJ, USA) and present combination indices in the Chou-Talalay plot [25], which allows us to determine whether the drug interaction shows CI < 1: synergistic effect; CI = 1: additive effect; CI > 1: antagonist effect.

### Statistical analysis

Data are presented as mean ± standard deviation. Comparisons among treatment groups were performed using one-way analysis of variance (ANOVA), followed by a Tukey post hoc test. A *p* value of < 0.05 was considered statistically significant.

## Results

### Optimization of the combination of BMX and TMZ in three CRC cell lines

To investigate the influence of BMX or TMZ on CRC cell growth, three human colorectal cancer cell lines, HT29 (p53 mutation), HCT116 (p53 wild-type), and RKO (p53 wild-type), were utilized. They were separately treated with BMX (0.313, 0.625, 1.25, 2.5, 5 and 10 μM) or TMZ (25, 50, 100, 200, 400, 800, and 1000 μM) for 24, 48, and 72 h. The results showed that CRC cell viability was inhibited significantly in a dose-dependent manner (Additional file 1: Figure S1A). The half-maximal inhibitory concentration (IC_50_) values of BMX or TMZ alone in the HT29, HCT116, and RKO cells were determined (Table 1). For the clonogenic assay, which represented in vivo tumorigenicity, TMZ was effective against tumor sphere formation in the clonogenic assay of the HT29, HCT116, and RKO cells, and the IC_50_ values of TMZ were 359.45 ± 50.43, 137.66 ± 22.73, and 244.01 ± 29.42 µM, respectively (Additional file 1: Figure S1B). The results show the basic cell proliferation inhibition rates of BMX and TMZ for the three incubation times in the three colorectal cancer cells, HT-29, HCT-116, and RKO.

**Table 1:** The half maximal inhibitory concentration (IC_50,_ μM) of different combinations with BMX and TMZ in three CRC cell lines

Combination and incubation time	HT29	HCT116	RKO
*BMX alone*^*a*^			
24 h	42.6 ± 2.4	24.8 ± 2.5	38.5 ± 3.5
48 h	9.9 ± 0.5	7.7 ± 0.3	7.2 ± 0.6
72 h	2.9 ± 0.2	1.5 ± 0.3	1.5 ± 0.3
*TMZ alone*^*b*^			
24 h	> 1000	> 1000	> 1000
48 h	930.8 ± 47.7	515.2 ± 21.6	991.6 ± 52.4
72 h	257.6 ± 20.53	190.0 ± 14.7	380.5 ± 40.3
*5 μM BMX* + *TMZ*^*c*^			
24 h	> 400	> 400	> 400
48 h	128.3 ± 18.3	41.56 ± 2.4	21.9 ± 2.7
72 h	NA	NA	NA
*50 μM TMZ* + *BMX*^*d*^			
24 h	> 10	> 10	> 10
48 h	9.1 ± 0.2	3.2 ± 0.3	3.6 ± 0.4
72 h	2.2 ± 0.1	0.9 ± 0.1	0.9 ± 0.1

^a^Treat indicated cell with BMX (0.5, 10, 15, 30 and 50 µM)

^b^Treat indicated cell with TMZ (25, 50, 100, 200, 400, 800, 1000 µM)

^c^Treat indicated cell with different concentrations of TMZ combination of 10 μM BMX

^d^Treat indicated cell with different concentrations of BMX combination of 50 μM TMZ

To evaluate whether BMX improved the chemosensitivity of TMZ, BMX and TMZ were administered together to treat HT29, HCT116, and RKO cells (Additional file 1: Figure S1C). The combination of BMX (5 µM) and TMZ (25, 50, 100, 200, and 400 µM) exhibited a greater inhibitory effect on cell growth than either BMX or TMZ alone. Subsequently, 50 μM TMZ combined with different concentrations of BMX (0.313, 0.625, 1.25, 2.5, 5, and 10 μM) suppressed cell proliferation in a time-dependent manner. Notably, BMX decreased the IC_50_ of TMZ in HT29, HCT116, and RKO cells (Table 1). These findings suggest that BMX inhibited CRC cell proliferation and improved the chemosensitivity of TMZ. Fifty μM TMZ with 5 μM BMX exerted the highest cytotoxic effect in HT29, HCT116, and RKO cells. We used this combination in a time-dependent manner and noted a cytotoxic effect at 48 h. This finding suggests that BMX improved the chemosensitivity of TMZ. BMX in combination with TMZ suppressed cell proliferation in a time-dependent manner. Thus, all subsequent experiments were performed using 50 μM TMZ combined with different concentrations of BMX (2.5, 5, and 10 μM) for 48 h. We next examined colony formation in the presence of BMX alone or combined with TMZ. In regular continuous fashion, we treated cells with BMX alone at concentrations of 5 to 10 μM in HT29, 2.5 to 5 μM in HCT116 and RKO and the results showed it induced an inhibitory effect (Additional file 1: Figure S1D). By Additional file 1: Figure S2 and Table 2, both 50 μM TMZ combines with indicated BMX (2.5, 5, and 10 μM) or 5 µM BMX combines with indicated TMZ (25, 50, 100, 200, and 400 µM) exhibits the strongest synergistic cytotoxicity effect in either 24, 48, and 72 h group. In addition, this inhibitory effect increased when combining BMX with 50 μM TMZ (Additional file 1: Figure S3). If TMZ was increased (150 μM), BMX could be lower down to 1–2 μM instead of 5–10 μM (Additional file 1: Figure S3), suggesting that reframed TMZ could be used as a potential repurposed drug or adjuvant for CRC therapy.

**Table 2:** BMX exhibits synergistic effect in combination with TMZ in CRC cells. Drug toxicity was determined via CCK-8 assay in HT29, HCT116 and RKO cells

Drug combinations		HT29		HCT116		RKO	
BMX (uM)	TMZ (uM)	CI^a^	Interpretation^b^	CI^a^	Interpretation^b^	CI^a^	Interpretation^b^
0.3125	50	0.31	++++	0.46	+++	0.33	++++
0.625		0.3	++++	0.45	+++	0.32	++++
1.25		0.35	++++	0.48	+++	0.36	++++
2.5		0.42	+++	0.46	+++	0.47	+++
5		0.57	+++	0.57	+++	0.67	++
10		0.42	+++	0.17	++++	0.60	++
5	25	0.98	+	0.79	++	0.75	++
	50	0.57	+++	0.53	+++	0.52	++
	100	0.43	+++	0.47	+++	0.46	+++
	200	0.39	++++	0.49	+++	0.26	++++
	400	0.29	++++	0.60	+++	0.27	++++

^a^CI < 1: synergistic effect; CI = 1: additive effect; CI > 1: antagonist effect

^b^CI 0.8–0.99: slight synergism (+); CI 0.6–0.8: moderate synergism (++); CI 0.4–0.6: synergism (+++); CI 0.2–0.4: strong synergism (++++)

Taken together, these results demonstrate the combined use of BMX and TMZ synergistically inhibits proliferation and colony formation of CRC cancer cells. Thus, all subsequent experiments were performed using 50 μM TMZ combined with different concentrations of BMX (2.5, 5, and 10 μM) for 48 h.

### The effects of the combination of BMX and TMZ compared with conventional drugs on CRC

**Fig. 1:**
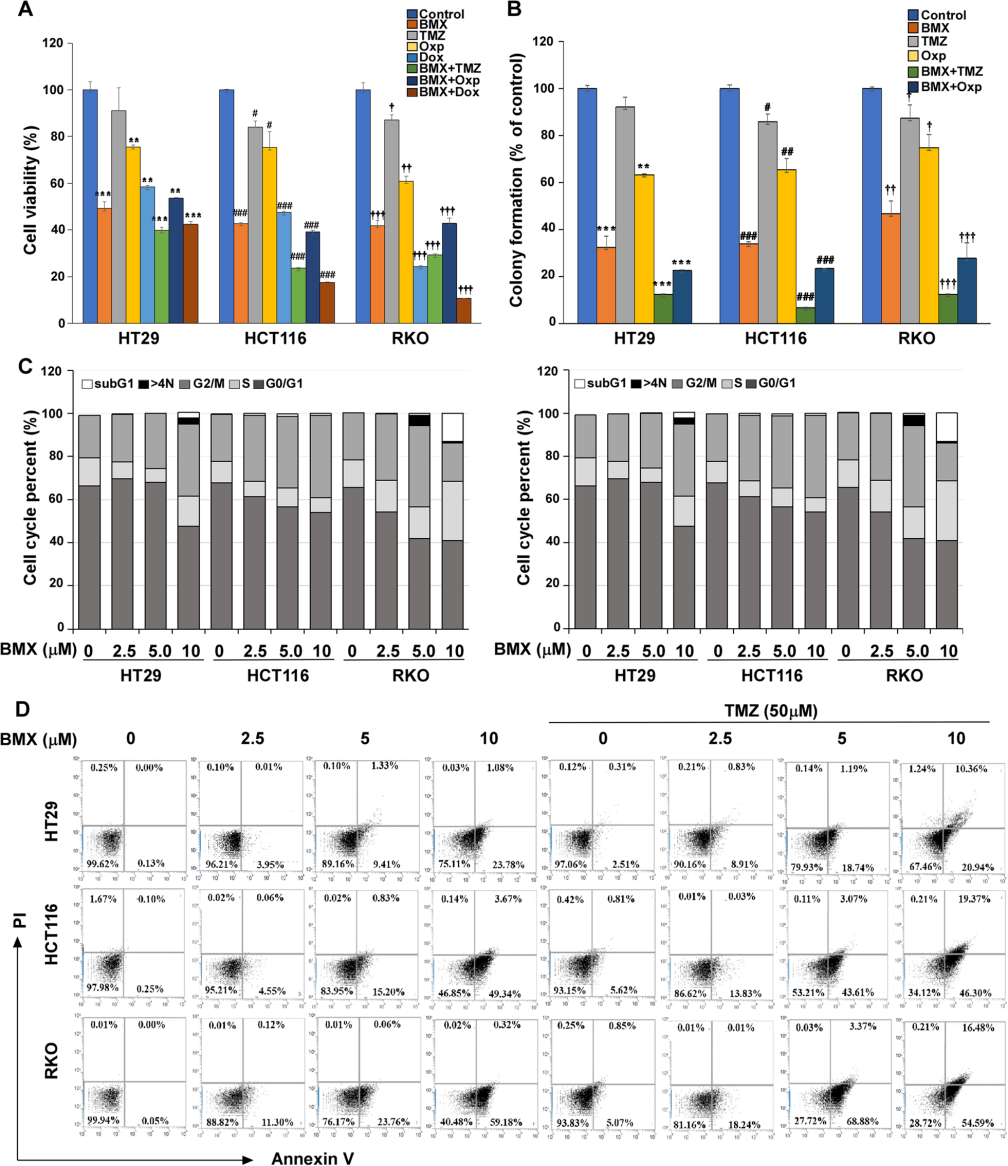
BMX, TMZ, oxaliplatin (Oxp) and doxorubicin (Dox) combination inhibited cell proliferation in CRC cells. (**A**) The proliferation of BMX, TMZ, Oxp, Dox, BMX plus TMZ, BMX plus Oxp or BMX plus Dox in HT29, HCT116 and RKO cells with various drug concentration. (**B**) Colony formation capability assay with different treatments of BMX, TMZ, Oxp, BMX plus TMZ, and BMX plus Oxp in HT29, HCT116 and RKO cells; the colonies were counted for quantification. (**C**) Cell cycle analysis after 48 h treatment with different concentrations of BMX alone or BMX combined with TMZ in HT29, HCT116, and RKO cells and the proportion of cells in each cell cycle phase. SubG1, cell with polyploid chromosome; > 4 N, polyploid cell. (**D**) Apoptosis analysis after 48 h treatment with different concentrations of BMX alone or BMX combined with TMZ and the apoptotic rate of cells in HT29, HCT116, and RKO cells. All results are shown as mean ± s.d. from three independent experiments. *p < 0.05, **p < 0.01, ***p < 0.001 versus control (HT29 cells); #*p* < 0.05, ##*p* < 0.01, ###*p* < 0.001 vs. control (HCT116 cells); †*p* < 0.05, ††*p* < 0.01, †††*p* < 0.001 versus control (RKO cells)

To further explore the simultaneous treatment of cells with different combinations of BMX and TMZ, we investigated whether addition of Oxp and Doxorubicin (Dox) could boost the cytotoxic effects of BMX. Hence, cells were incubated with BMX (10 μM) and TMZ (50 μM), DOX (1 μM) or Oxp (5 μM) for 48 h. We found that BMX could potentiate cytotoxicity induced by chemotherapeutic agents. Moreover, the combination of Oxp or Dox with BMX against HT29, HCT116, and RKO cells suppressed cell proliferation (Fig. 1A and B). In addition, all of these drugs were effective in inhibiting tumor sphere formation in the clonogenic assay, which represents tumorigenicity, in HT29, HCT116, and RKO cells. Combination of BMX and TMZ treatment showed the highest anti-clonogenic growth compared with the other groups in the three cell lines. However, a combination of BMX and Dox in RKO showed survival rate of less than 10% and stopped growth in the anti-clonogenic assay. Although TMZ plus BMX was not better than Dox plus BMX, TMZ plus BMX still was better than Oxp, which is used in conventional CRC treatment (Fig. 1A and B).

Cell cycle arrest is the most common cause of inhibition of cell proliferation. Possible mechanisms by which BMX treatment alone or in combination inhibited cell proliferation and cell cycle profile were assessed by flow cytometry analysis. As shown in Fig. 1C, Additional file 1: Figure S4A, and S4B, 2.5, 5, and 10 μM BMX treatment significantly increased the percentage of cells in the G2/M phase in HT29 and HCT116 cells and significantly decreased the percentage of cells in the sub G1 phase (apoptosis) in RKO cells. Notably, BMX plus TMZ treatment dramatically induced G2/M phase arrest in HT29 cells, and increased the sub G1 phase (apoptosis) in HCT116 and RKO cells.

The synergistic effect of BMX with TMZ after treatment for 48 h was measured by Annexin V binding in three CRC cells. Treatment of cells with BMX in combination with TMZ resulted in a marked increase in the proportion of apoptotic cells compared with that of BMX or TMZ alone (Fig. 1D). BMX increased early apoptotic cells up to 23.78%, 49.34%, and 59.18% in HT29, HCT116, and RKO cells, as did the late apoptosis. The combination treatment resulted in increased populations of late apoptosis from 1.08 to 10.36%, 3.67 to 19.37%, and 0.32 to 16.48% in HT29, HCT116, and RKO cells, respectively, after 48 h incubation (Fig. 1D).

### Induction of apoptosis by BMX and combined BMX plus TMZ was mediated by p53-dependent MGMT inhibition

**Fig. 2:**
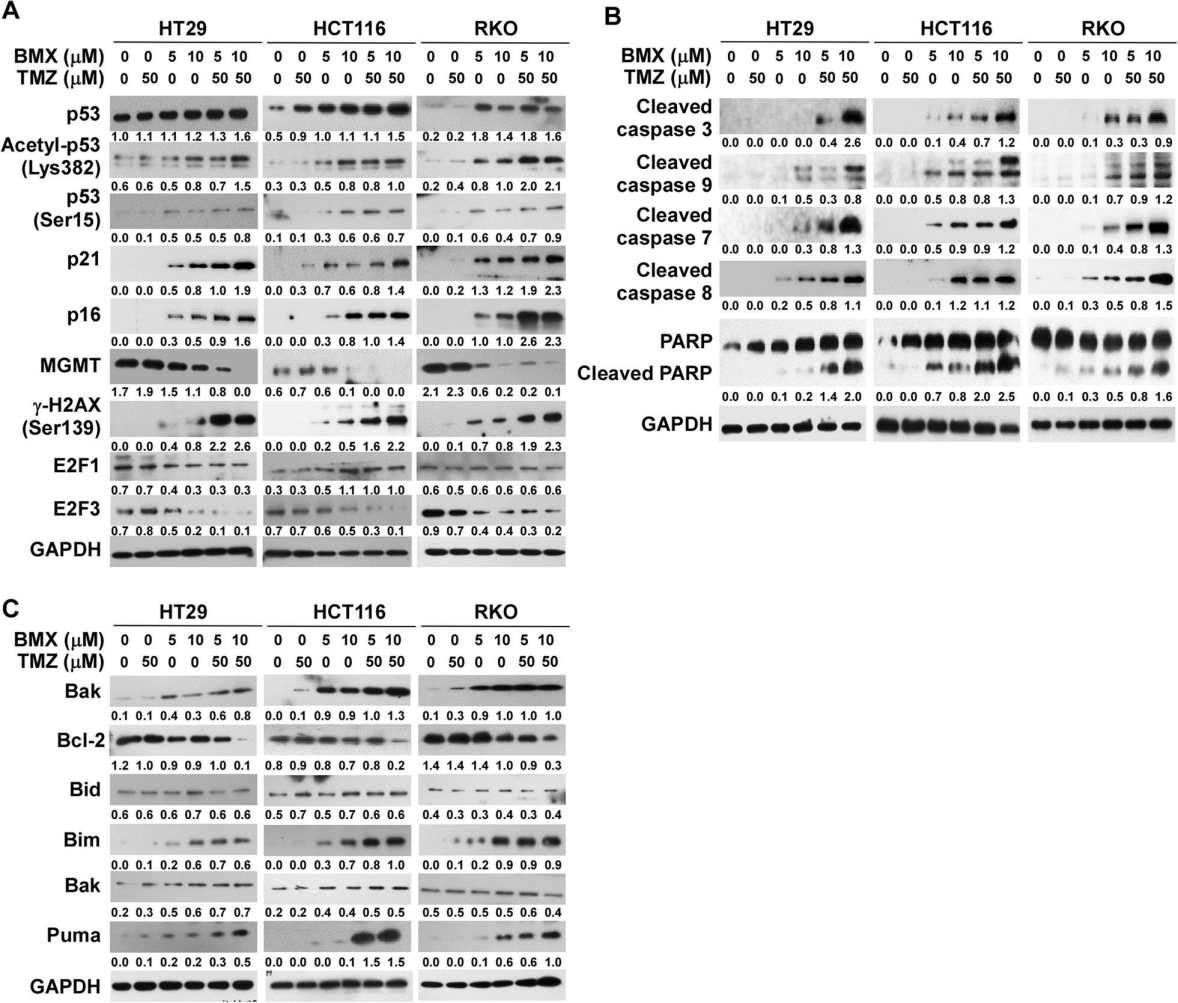
BMX and BMX plus TMZ combination-induced apoptosis and autophagy were mediated by p53-mediated MGMT inhibition. (**A**) Western blot analysis of p53, p53 Lys382 acetylation, p53 Ser15 phosphorylation, p21, p16, MGMT, γ-H2AX, E2F1, and E2F3 expression in HT29, HCT116, and RKO cells treated with various concentrations of BMX (5 and 10 μM) and BMX combined with TMZ for 48 h. (**B**) Expressions of cleaved caspase 3, 7, 8, 9, and cleaved PARP proteins in HT29, HCT116, and RKO cells treated with various concentrations of BMX (5 and 10 μM) and BMX combined with TMZ for 48 h. (**C**) Expressions of Bax, Bcl-2, Bid, Bim, Bak, and Puna proteins in HT29, HCT116, and RKO cells treated with various concentrations of BMX (5 and 10 μM) and BMX combined with TMZ for 48 h. GAPDH was used as the loading control

BMX has been shown to activate p53, which is involved in cell death of various cancer cells induced by chemotherapy drugs, and this is mediated by the β-catenin pathway [8, 26]. To verify whether the anticancer activities of BMX with TMZ were partly due to DNA damage, we examined the extent of DNA damage and the changes in the p53 pathway markers in response to DNA damage caused by BMX plus TMZ in three CRC cell lines. The basic protein expression status of markers in HT29, HCT116, and RKO cells is shown (Additional file 1: Figure S5). The BMX alone group showed increased p53 protein level, which caused cell cycle arrest and inhibited cell growth. Treatment with BMX alone or BMX plus TMZ combination dose-dependently increased the levels of p53 phosphorylation (Ser15) and γ-H2AX phosphorylation (Ser139) in HT29, HCT116, and RKO cells. In the HT29, HCT116, and RKO cells, acetylation of p53 at Lys382 increased in a time-dependent manner, and enhanced expression of p53 downstream target p21 and p16. Moreover, by evaluating the Western blotting for wild-type p53 (HCT116 and RKO) and mutant (HT29) cells, it was found that CRC wild-type p53 cells are MGMT hypermethylated and lower the MGMT protein expression as well. In addition, the BMX plus TMZ combination significantly decreased E2F3, but not E2F1 expression (Fig. 2A). Interestingly, acetylation of histone H3 and H4 was also increased by BMX alone or BMX plus TMZ (Additional file 1: Figure S6). This suggests that BMX affects the activity of histone acetyltransferases and/or HDACs. Combined BMX plus TMZ can increase p21, p16 expression, and γ-H2AX phosphorylation through enhancement of p53 expression and activation of p53-mediated MGMT inhibition (Fig. 2A).

The balance between pro‐apoptotic (stress or death) signals and anti‐apoptotic molecules, including Bcl-2 and Bid, Bax or Puma, is the main cause of the apoptotic response through the caspase-dependent pathway [27]. The cleavage of caspases, depicted in Fig. 2B, suggests that caspase 7, caspase 8, caspase 9, and caspase 3 activities were not significantly changed by BMX at lower concentrations, although when combined with TMZ in HT29 cells, they were highly upregulated in a dose-dependent manner, contributing to PARP cleavage and apoptosis eventually. Apoptosis protein expression levels of cleaved caspase 3, caspase 7, caspase 8, caspase 9, and PARP were found to significantly increase in a concentration-dependent manner following BMX 10 μM treatment in the HCT116 and RKO cell lines. Moreover, we investigated the pro-apoptotic and anti-apoptotic signals in wild-type p53- or mutant p53-mediated apoptosis. The results revealed that the BMX treatment decreased the level of the antiapoptotic proteins Bcl-2 and increased proapoptotic proteins Bax, Bim, and Puma. However, BMX treatment did not lead to the upregulation of the proapoptotic Bcl-2 family proteins Bak and Bid. In addition, the synergistic effect of BMX and TMZ was better than BMX alone (Fig. 2C). The combination of TMZ plus BMX resulted in more senescent cells than each treatment alone, especially in wild-type p53 cells, such as HCT116 and RKO (Additional file 1: Figure S7). Because CD133, CD44, and SOX2 are highly related to the drug resistance of CSCs and are used as phenotypic markers for CSC including CRC, treatment with BMX plus TMZ was able to reduce the gene expression levels of CD133, CD44 and SOX2 in a dose-dependent manner in HT29, HCT116, and RKO (Additional file 1: Figure S8). Thus, the BMX and TMZ combination attenuated CSC markers, which resulted in the TMZ-mediated cytotoxic effect enhancement. Therefore, the results above indicate that the caspase-dependent signaling pathway was activated by the BMX and TMZ combination treatment in CRC cells to induce cell apoptosis. In addition, BMX plus TMZ combination-induced apoptosis was mediated by p53-dependent MGMT inhibition.

### BMX enhanced TMZ-mediated cytotoxic activity through the Wnt/β-catenin pathway

**Fig. 3:**
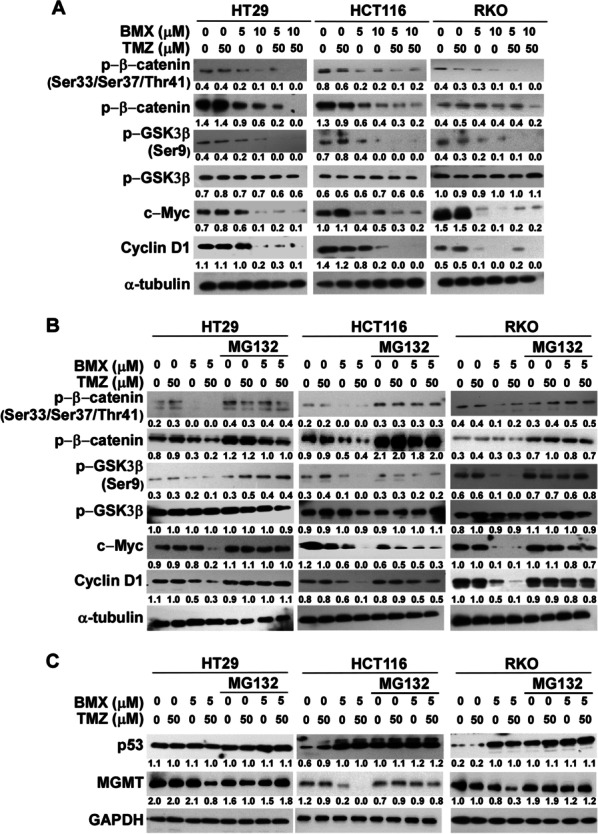
BMX enhanced TMZ to cause a cytotoxic effect on CRC cells through the Wnt/β-catenin pathway. (**A**) GSK3β, β‑catenin activation status, c-Myc and Cyclin D1 of HT29, HCT116, and RKO cells after treatment with BMX with or without TMZ for 48 h. (**B**) GSK3β, β‑catenin activation status, c-Myc and Cyclin D1 expression were upregulated by BMX with or without TMZ and MG132 in T29, HCT116, and RKO cells. (**C**) p53 and MGMT expression was upregulated by BMX with or without TMZ and MG132 in HT29, HCT116, and RKO cells. GAPDH was used as the loading control

We investigated the mechanisms of combined treatment-induced cytotoxic effect in three CRC cell lines. Our GSEA revealed that upregulated ranked genes in both HT29 and RKO cells by BMX treatment were highly similar to genes upregulated after CTNNB1 (β-catenin encoding gene) deletion. It is possible that BMX might decrease β-catenin in CRC cells (Additional file 1: Figure S9A). As shown in Fig. 3A, β-catenin, phospho-β-catenin (Ser33/Ser37/Thr41), and phospho-GSK3β (Ser9) protein expression levels were increased, while phospho-β-catenin (Ser33/Ser37/Thr41) and phospho-GSK3β (Ser9) levels were decreased by BMX treatment in three CRC cell lines. Combined treatment with 5–10 μM BMX and 50 μM TMZ reduced β-catenin protein levels and decreased the protein levels of phospho-β-catenin (Ser33/Ser37/Thr41) through phosphorylation by GSK3β in three cell lines. Furthermore, we further examined the effects of BMX on proliferation and noted that BMX, both with and without TMZ, could decrease proliferative markers c-Myc and cyclin D1 (Fig. 3A). The combined treatment with 5 μM BMX and TMZ enhanced GSK3β-mediated Ser9 phosphorylation, further increased the GSK3β phosphorylation at Ser33/Ser37/Thr41, and eventually caused β-catenin degradation (Fig. 3B). In addition, MG132 application reversed β-catenin degradation and increased MGMT expression under 5 μM BMX and 50 μM TMZ (Fig. 3C). Taken together, these data revealed that BMX enhanced TMZ-mediated cytotoxic activity, partly via the Wnt/β-catenin pathway, thus reducing CRC cell proliferation.

### Autophagy served as a key regulator in BMX- and BMX plus TMZ combination-induced cell death

**Fig. 4:**
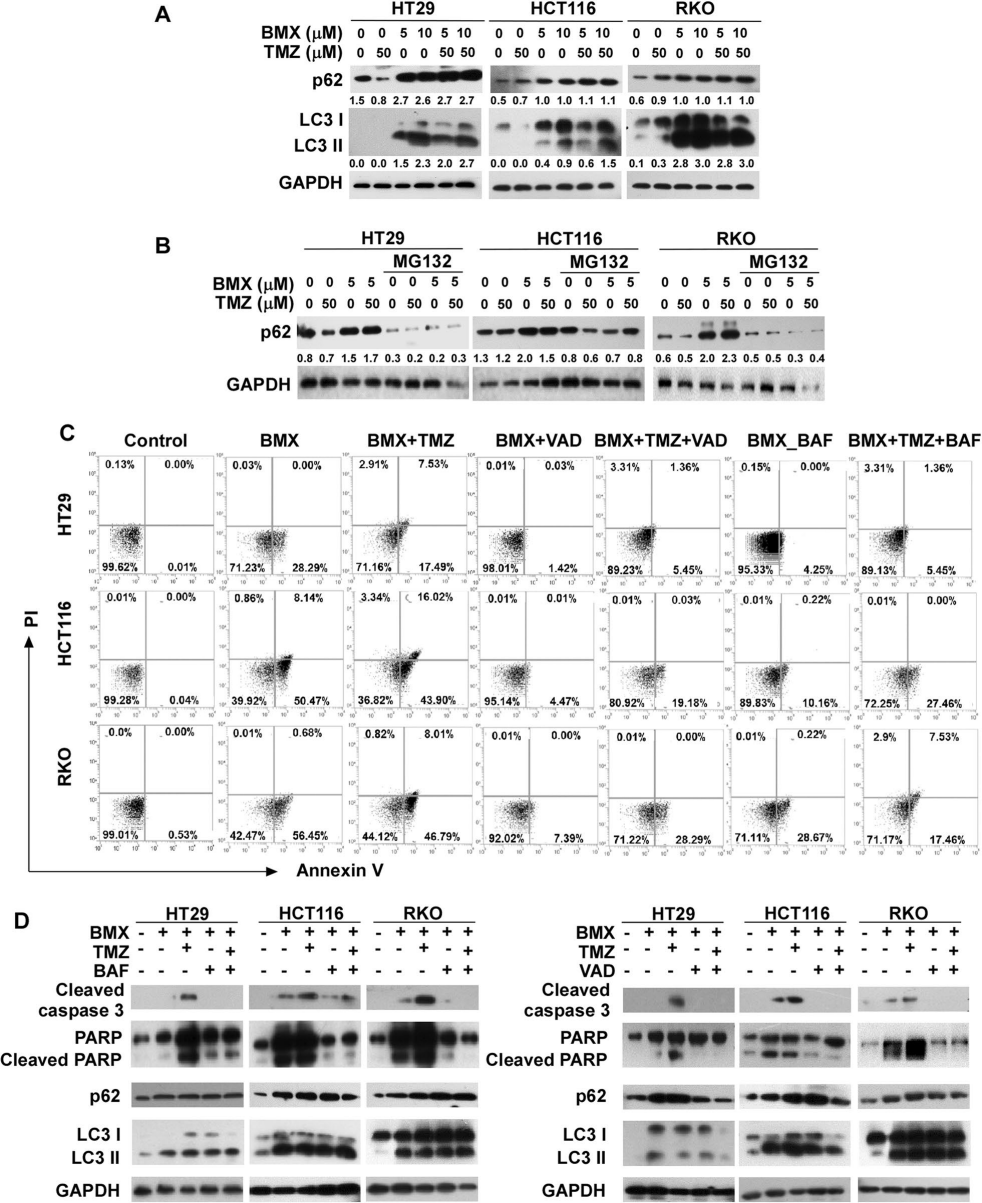
Autophagy was responsible for cell death induced by BMX alone and BMX plus TMZ combination. (**A**) LC3 and p62 expression in HT29, HCT116, and RKO cells treated with BMX (5 and 10 µM) with or without TMZ evaluated by Western blot. (**B**) p62 expression was downregulated by BMX with or without TMZ and MG132 in HT29, HCT116, and RKO cells. (**C**) Pre-treatment with BAF and VAD reduced the cell apoptosis in HT29, HCT116, and RKO cells exposed to BMX with or without TMZ for 48 h. (**D**) Effects of VAD and BAF on BMX with or without TMZ induced cleaved caspase 3, cleaved PARP, p62, and LC3 expression. GAPDH was used as the loading control

Lipidated LC3 and autophagy substrate p62 are frequently used as markers to assess autophagosomes and autophagy [9]. Treatment with BMX alone or combined treatment with BMX and TMZ also yielded a dose-dependent increase in the expression of LC3-II and p62 (Fig. 4A). As previously reported, β-catenin negatively regulates p62 expression [9, 28]. Therefore, we used proteasome inhibitor MG132 to evaluate whether suppressed β-catenin protein degradation can trigger p62 protein upregulation in cells treated by BMX alone or BMX plus TMZ combination. The result showed that p62 protein level was indeed upregulated when MG132 was applied (Fig. 4B). Due to β-catenin protein degradation with combination treatment, p62 was no longer inhibited, which then triggered the downstream autophagy pathway (Fig. 4B). To determine the role of autophagy in BMX alone or BMX and TMZ combination-induced cell death, we used BAF and Z-VAD-FMK (carbobenzoxy-valyl-alanyl-aspartyl-[O-methyl]-fluoromethylketone), to treat cells before addition of BMX alone or BMX plus TMZ combination, and we found that Z-VAD-FMK suppressed early apoptosis induced by BMX plus TMZ treatment in the three cell lines Fig. 4C). In addition, pre-treatment with BAF, BMX alone or BMX and TMZ combination also reduced caspase 3, caspase 7, caspase 8, and caspase 9 cleavage (Fig. 4D). The apoptosis and autophagy related proteins and the corresponding signaling pathways have been identified, implying crosstalk between autophagy and apoptosis [29]. To test the involvement of apoptosis in BMX-induced autophagy, we treated cells with BAF or Z-VAD-FMK before adding BMX alone or BMX plus TMZ combination. As shown in Fig. 4D, although Z-VAD-FMK and BAF showed early apoptosis suppression, BAF inhibited BMX plus TMZ combination-induced caspase 3 activation without interfering with LC3I/II in all cells. However, Z-VAD-FMK inhibited BMX plus TMZ combination-induced caspase 3 activation with interference of LC3 I/II in the mutant-type p53 cell line. Taken together, these results suggest that autophagy stimulation is an important pathway by which apoptosis promotes cell death.

### Evaluation of HDAC cytotoxicity with broad-spectrum inhibitors

**Fig. 5:**
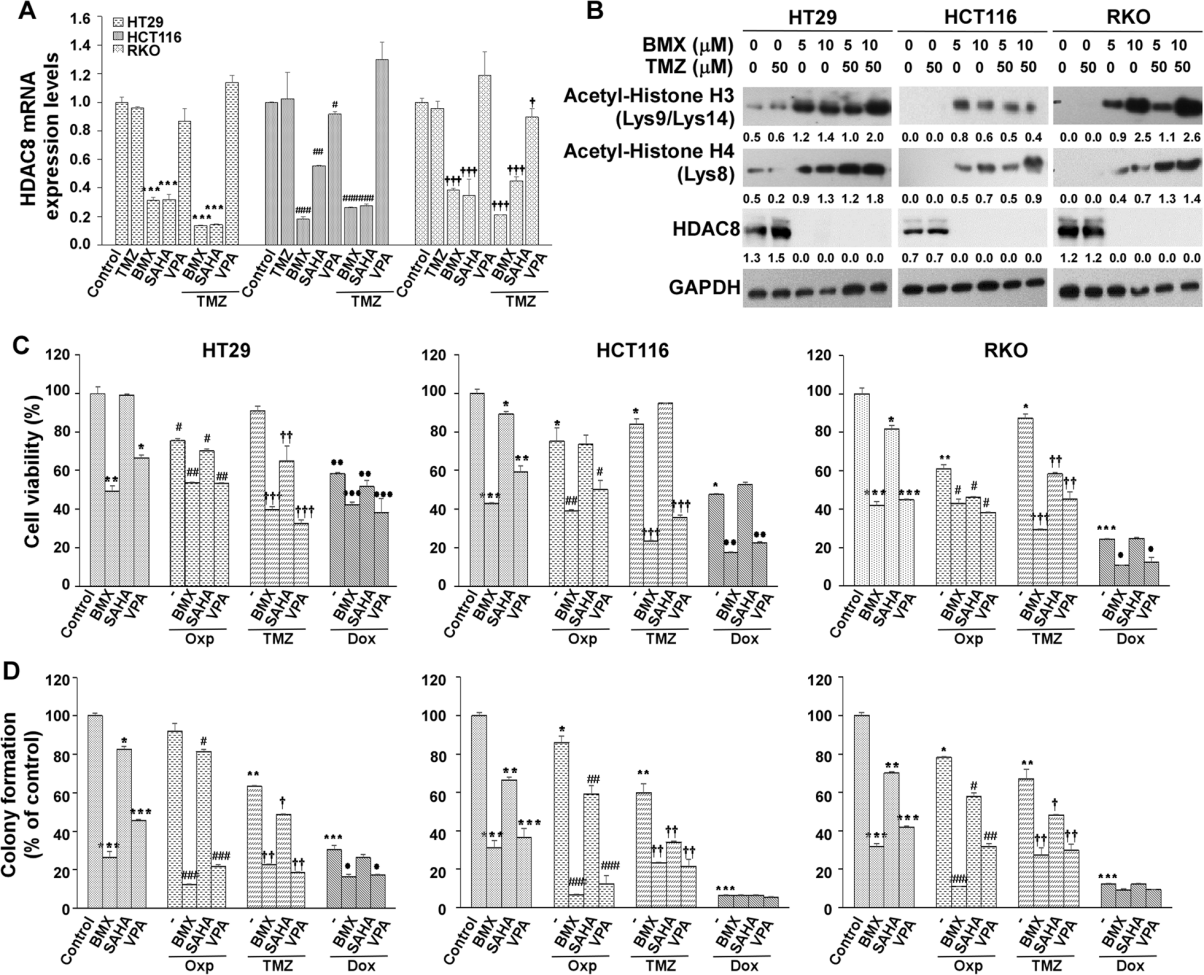
BMX, VPA, SAHA, TMZ, Oxp, and Dox combination inhibited cell proliferation in CRC cells. (**A**) HDAC8 mRNA expression levels stimulated with BMX (5 µM), VPA (4 mM), SAHA (2 µM) with or without TMZ were determined using qRT-PCR assays. (**B**) HT29, HCT116, and RKO cells were treated with BMX with or without TMZ for 48 h. Then, cells were harvested for detection of acetyl-histone H3 (Lys9/Lys14), acetyl-histone H4 (Lys8), and HDAC8 by Western blot analysis. (**C**) The proliferation of BMX, VPA, SAHA with or without TMZ, Oxp (5 µM) and Dox (1 µM) in HT29, HCT116, and RKO cells with treatment durations were assayed using the CCK-8 method. (**D**) Colony formation capability assay with different treatments of BMX, VPA, SAHA with or without TMZ, Oxp and Dox in HT29, HCT116, and RKO cells; the colonies were counted for quantification. All results are shown as mean ± s.d. from three independent experiments. *p < 0.05, **p < 0.01, ***p < 0.001 versus control (HT29 cells); #*p* < 0.05, ##*p* < 0.01, ###*p* < 0.001 versus control (HCT116 cells); †*p* < 0.05, ††*p* < 0.01, †††*p* < 0.001 versus control (RKO cells)

To examine if BMX alone or BMX plus TMZ combination could promote autophagy during cell death in HT29, HCT116, and RKO cells, first we applied BMX, an inhibitor of HDAC8, to analyze the mRNA and protein level of HDAC8. To further determine whether histone deacetylation 8 is implicated in cell death, two different HDAC inhibitors (SAHA and VPA) were used to investigate the mechanism of cell death in HT29, HCT116, and RKO cells. HDAC8 mRNA was assessed in HT29, HCT116, and RKO cells treated with 10 µM BMX, 2 µM SAHA, 4 mM VPA and 50 µM TMZ. The results indicated that BMX and SAHA HDACi downregulated HDAC8 in HT29, HCT116, and RKO cells. Notably, VPA did not decrease HDAC8 mRNA expression like the other HDACi did (Fig. 5A). A significant decrease in HDAC8 levels was observed in HT29, HCT116, and RKO cells after BMX, SAHA, BMX plus TMZ combination or SAHA plus TMZ combination treatment. In addition, acetylation of histone H3 and histone H4 was also increased by BMX, SAHA, BMX plus TMZ combination, or SAHA plus TMZ combination (Fig. 5B). Therefore, we speculate that HDAC8 may serve a key role in the regulation of cell death in HT29, HCT116, and RKO cells.

BMX and SAHA significantly reduced the growth of HT29, HCT116, and RKO cells (Figure S10A, S10B and S10C). It is worth noting that VPA alone or in combination with TMZ in the three cell lines did not show significant cytotoxicity. Dox showed significant inhibitory effects on survival in HCT116 and RKO cells, especially combined with BMX or SAHA. However, when compared with Oxp, TMZ in combination with BMX or SAHA showed a cytotoxic advantage compared with TMZ alone (Fig. 5C, D and Additional file 1: Figure S10D). Therefore, TMZ plus BMX treatment had the highest cytotoxicity compared with the other groups in the three cell lines.

### TMZ-mediated cytotoxic effects were enhanced by promoting TMZ-mediated apoptosis in CRC cells under BMX treatment

**Fig. 6:**
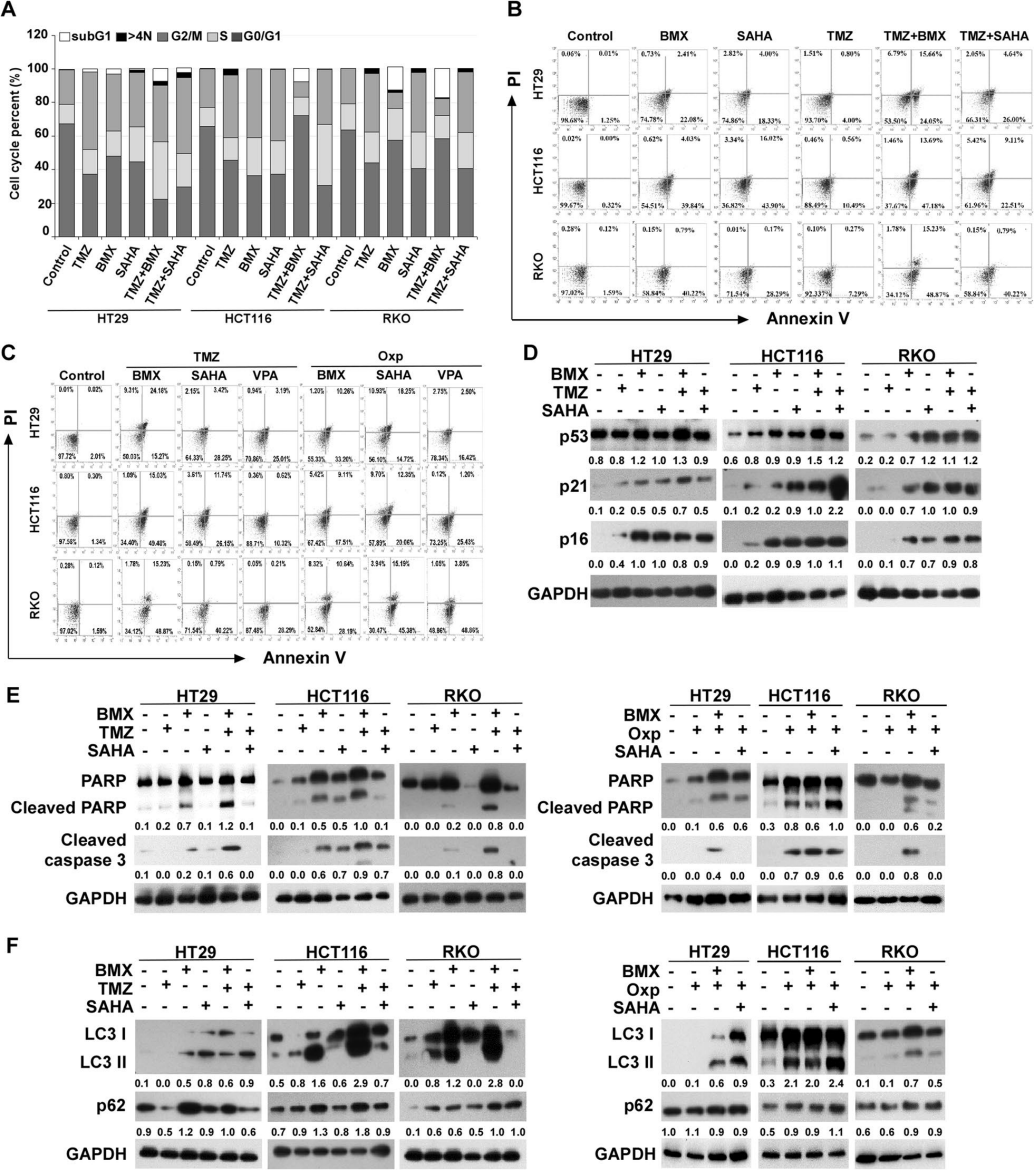
BMX but not SAHA synergistically enhances the cytotoxic effects of TMZ. (**A**) Cell cycle analysis after 48 h treatment with BMX and SAHA with or without TMZ in HT29, HCT116, and RKO cells, and the proportion of cells in each cell cycle phase. SubG1, cell with polyploid chromosome; > 4 N, polyploid cell. (**B**) Apoptosis analysis after 48 h treatment BMX and SAHA with or without TMZ and the apoptotic rate of cells in HT29, HCT116, and RKO cells. (**C**) Apoptosis analysis after 48 h treatment with BMX, VPA, or SAHA with TMZ, and Oxp and the apoptotic rate of cells in HT29, HCT116, and RKO cells. (**D**) Western blot analysis of p53, p21, and p16 expression in HT29, HCT116, and RKO cells treated with BMX and SAHA with or without TMZ for 48 h. (**E**) Expressions of cleaved caspase 3 and cleaved PARP proteins in HT29, HCT116, and RKO cells treated with BMX and SAHA with or without TMZ for 48 h. (**F**) Expressions of LC3 and p62 proteins in HT29, HCT116, and RKO cells treated with BMX and SAHA with or without TMZ for 48 h

SAHA, VPA, TMZ, Dox, and Oxp are all drugs that form damage adducts on DNA [8, 30–32]. We therefore checked whether the drug treatments resulted in cell death via apoptosis. We observed both BMX alone and SAHA alone induced G2/M arrest in HT29, HCT116, and RKO cell lines. However, in HT29 and RKO cells, the significantly induced sub G1 phase arrest (apoptosis) of BMX alone was stronger than that of SAHA alone. In addition, the cytotoxic effect of BMX plus TMZ combination was better than that of the SAHA plus TMZ combination (Fig. 6A and Additional file 1: Figure S4C). Both BMX alone and SAHA alone promoted early apoptosis, especially in RKO cells. Moreover, BMX plus TMZ combination showed better late apoptosis than that of SAHA plus TMZ combination (Fig. 6B). We therefore checked whether the drug treatments resulted in cell death via apoptosis. To systematically identify active drug combinations, we used the cell cycle and apoptosis to measure the effects of SAHA, VPA, BMX, TMZ, Dox, and Oxp drug screening combinations on HT29, HCT116, and RKO cells. Oxp combined with BMX, SAHA, or VPA could promote early apoptosis in all cell lines, whereas TMZ combined with BMX, SAHA, or VPA only worked on HCT116 (wild-type p53) and RKO (wild-type p53). In HT29 cells (p53 mutation), the apoptosis effect induced by Oxp plus BMX was better than that of TMZ plus BMX. However, in HCT116 and RKO cells, the effect of TMZ plus BMX was superior to that of the other combinations. Furthermore, TMZ plus BMX treatment had the highest percentage of late apoptosis among the other combinations (Fig. 6C). Collectively, our data suggest that TMZ-mediated cytotoxic effects were enhanced by promoting TMZ-mediated apoptosis in CRC cells under BMX treatment, but SAHA treatment showed little enhancement.

We further examined whether the effects of TMZ were mediated through mechanisms similar to those induced by HDACi. SAHA induced more p21 than BMX in HT29 cells (p53 mutant), but the reverse relationship was found in HCT116 and RKO cells (wild-type p53). TMZ plus BMX and TMZ plus SAHA enhanced p53 expression in HCT116 (wild-type p53) and RKO (wild-type p53). TMZ plus BMX and TMZ plus SAHA enhanced p21 and p16 expression in the three cell lines (Fig. 6D). TMZ plus BMX induced greater enhancement of caspase 3 and PARP cleavage than TMZ plus SAHA in HT29, HCT116, and RKO cells. Oxp combined with BMX enhanced greater caspase 3 and PARP cleavage than Oxp plus SAHA in all cell lines (Fig. 6E). In addition, SAHA did not enhance LC3 I/II after treating with TMZ in HCT116 and RKO cell (wild-type p53). TMZ plus BMX induced autophagy better than TMZ plus SAHA. Additionally, Oxp plus BMX induced more LC3II than TMZ plus BMX in HT29 cell (p53 mutation), but not in RKO (wild-type p53). Oxp without BMX or SAHA affected p62 expression (Fig. 6F), which indicated the synergistic autophagy induction ability of the TMZ plus BMX combination better than in all other combinations.

### Evaluation of the cytotoxicity of specific HDAC inhibitor

**Fig. 7:**
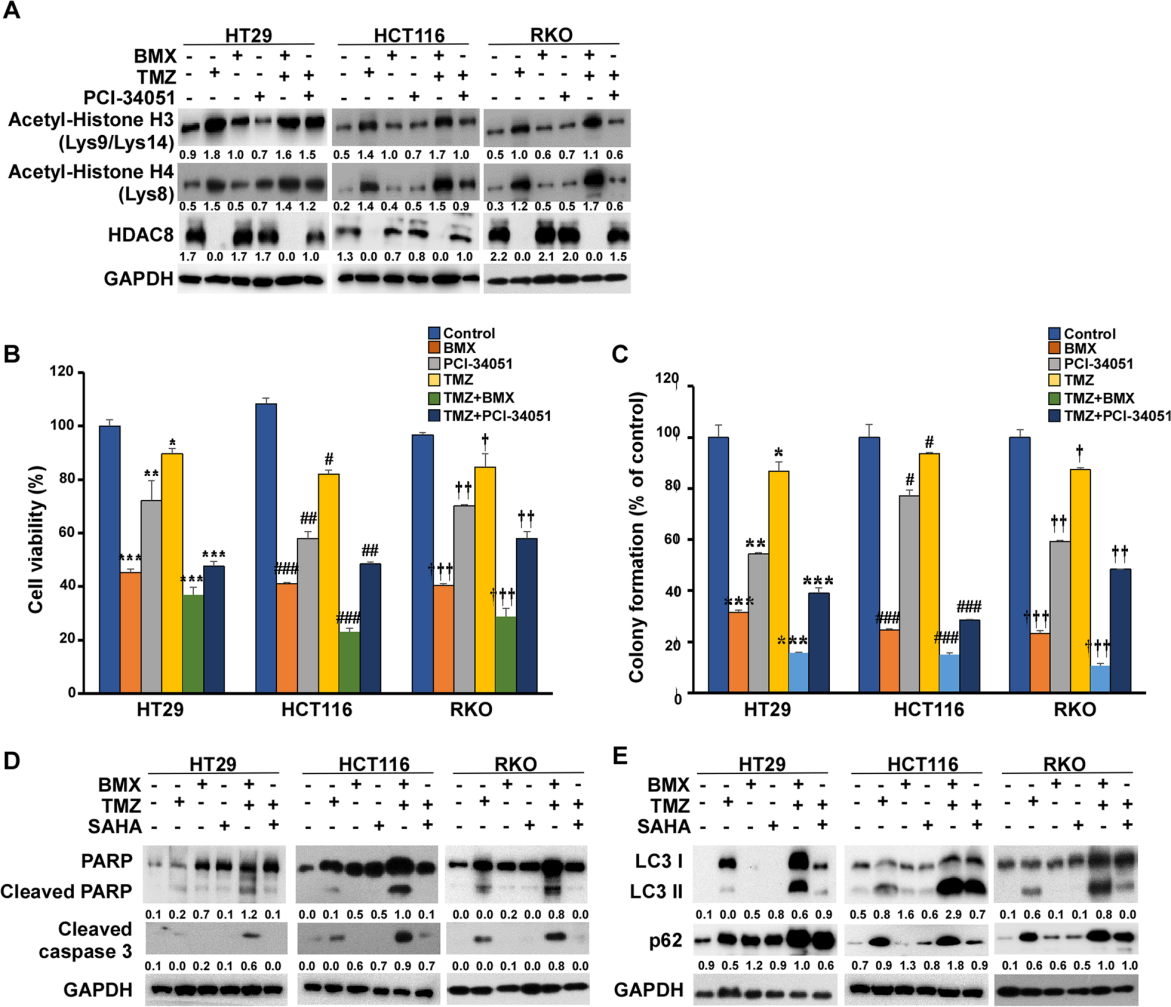
Autophagy expression levels stimulated with BMX and PCI-34051 in the presence or absence of TMZ. (**A**) HT29, HCT116, and RKO cells were treated with BMX and PCI-34051 with or without TMZ for 48 h. Then, cells were harvested for detection of acetyl-histone H3 (Lys9/Lys14), acetyl-histone H4 (Lys8), and HDAC8 by Western blot analysis. (**B**) The proliferation of BMX and PCI-34051 with or without TMZ in HT29, HCT116, and RKO cells. (**C**) Colony formation capability assay with different treatments of BMX and PCI-34051 with or without TMZ in HT29, HCT116, and RKO cells; the colonies were counted for quantification. (**D**, **E**) Expressions of cleaved caspase 3, cleaved PARP, LC3 and p62 proteins in HT29, HCT116, and RKO cells treated with BMX and PCI-34051 with or without TMZ for 48 h. All results are shown as mean ± s.d. from three independent experiments. *p < 0.05, **p < 0.01, ***p < 0.001 versus control (HT29 cells); #*p* < 0.05, ##*p* < 0.01, ###*p* < 0.001 versus control (HCT116 cells); †*p* < 0.05, ††*p* < 0.01, †††*p* < 0.001 versus control (RKO cells)

PCI-34051 has been evaluated in a preclinical trial as an HDAC8-selective inhibitor and has been shown to effectively induce caspase-dependent apoptosis^32^. Consistent with previous studies [33], we found that both BMX and PCI-34051 effectively induced H3 and H4 histone acetylation. However, PCI-34051 failed to induce inhibition of HDAC8 protein expression (Fig. 7A). In addition, the cell inhibition of cell proliferation and anti-clonogenic growth in TMZ plus BMX was stronger than TMZ plus PCI-34051 in HT29, HCT116, and RKO cells (Fig. 7B, C, Additional file 1: Figure S11). BMX was superior to PCI-34501 both in respect to apoptosis and autophagy activation (Fig. 7D and E). These results suggest that PCI-34051, despite its inability to inhibit HDAC8 protein expression, can induce autophagy activation. Of note, caspase-induced apoptosis is regarded as the major mechanism underlying HDAC8 inhibitor-induced cell death.

## Discussion

CRC treatments using traditional radio-chemotherapy are sometimes inefficient, partly because many CRC patients do not respond to this therapy regimen and/or suffer from severe drug toxicities. In the current study, we found more specific and efficient, synergistic apoptosis and anti-proliferative effects against CRC cells, particularly in the HCT116 and RKO cell lines, were achieved using a combination of BMX plus TMZ. Previous studies have shown that CRC patients could safely receive the combination treatment of FDA-approved anti-CRC drugs, such as fluoropyrimidine (5-FU), Oxp, irinotecan (IRI), and capecitabine (CAP or XELODA or XEL), with a response rate of approximately 20% [4, 5]. Nevertheless, there is growing evidence indicating that a combination therapy may be more effective than monotherapy in most malignancies [4, 5]. Two studies showed that TMZ either combined with PARP inhibitor or whole brain radiation can effectively inhibit the progression of CRC [34, 35]. However, in a study of 41 MGMT promoter methylated CRC patients, treatment with TMZ alone did not seem to have any promising clinical benefit [15]. Clearly, the role of TMZ in CRC is still controversial. Of note, the inhibitory effect increased when BMX was combined with 50 μM TMZ, and when TMZ dose was increased to 150 μM, the BMX could be lowered to 1–2 μM instead of 5–10 μM (Additional file 1: Figure S3), suggesting that BMX combined with TMZ cotreatment could be a useful precision medicine modality for CRC [12]. In fact, a recent clinical trial successfully proved the immune-sensitizing role of TMZ in patients with microsatellite stable; MGMT silenced metastatic CRC [36]. We propose that combining BMX with reframed TMZ as an adjuvant or repurposed drug may have some advantages for all subtypes of CRC patients [12, 16]. Therefore, it is crucial to identify the best synergistic drug and its mechanism. In the present study, we found that BMX, a specific HDAC8i, combined with TMZ induced cell cycle arrest, cell senescence, autophagy and apoptosis and inhibited cell proliferation, resulting in cell death.

BMX plus TMZ combination treatment induced synergistic apoptotic cell death, which was confirmed through caspase 3 cleavage and PARP activation (Fig. 2). The Wnt/β-catenin pathway has been shown to undergo aberrant activation in CRC [37]. In our previous work, BMX could overcome TMZ resistance in GBM. Our work demonstrates that the combination of BMX with TMZ may also have a beneficial clinical effect [8]. This combination treatment also resulted in cell proliferation and cell death by downregulating the Wnt/β-catenin pathway (Fig. 3). Treatment of CRC with HDACi induced γ-H2AX and activated the p53/p21 pathway, which led to apoptosis [38–40]. HDAC8 appears to be an attractive target for anticancer drug development since HDAC8 plays an explicit tumor-relevant role in CRC [41]. The knockdown of HDAC8 significantly increased expression and acetylation of p53 in cancer cells, resulting in decreased cell proliferation and increased apoptosis [22, 23]. Of note, for epigenetic players, in addition to its modifying effect on H3 and H4, BMX functions as an eraser inhibitor resulting in increased non-histone reader p53 (acetylation of p53) (Fig. 2) [24]. We conclude that BMX, a highly specific inhibitor of HDAC8, has utility as an epigenetic tool when used as a single agent and in combination with reframed TMZ to generate synergic effects. Hence, BMX appears to be a promising therapeutic target in personalized medicine for various CRCs. Moreover, we demonstrated that BMX combined with TMZ induced augmentation of phospho-p53 (ser15) as well as DNA damage, such as increased γ-H2AX foci (Fig. 2). Previous studies have reported that increased expression of phospho-p53 (ser15) is important for p53-mediated gene expression [8, 9, 42]. Moreover, the present study showed that the BMX plus TMZ combination exerted an HDAC-dependent synergistic effect on the viability of CRC cells.

The tumor suppressor protein p53 has been demonstrated to be implicated in the control of both apoptosis and autophagy by the upregulation of pro-apoptotic genes [43]. Autophagy is a highly conserved process that serves to induce cell death by degradation in the cytoplasm [44]. Numerous studies have shown that apoptosis and autophagy are interconnected in response to various anti-cancer therapeutics [9, 43, 45–49]. Previous studies showed that Thioridazine combined with TMZ triggers autophagy by enhancing p62 expression, which leads to subsequent inhibition of the Wnt/β-Catenin signaling pathway, as well as promotion of apoptosis in cancer cells [9]. Moreover, numerous studies have shown that there are certain crosstalk pathways among autophagy, apoptosis, and the Wnt/β-catenin pathway [46].

**Fig. 8:**
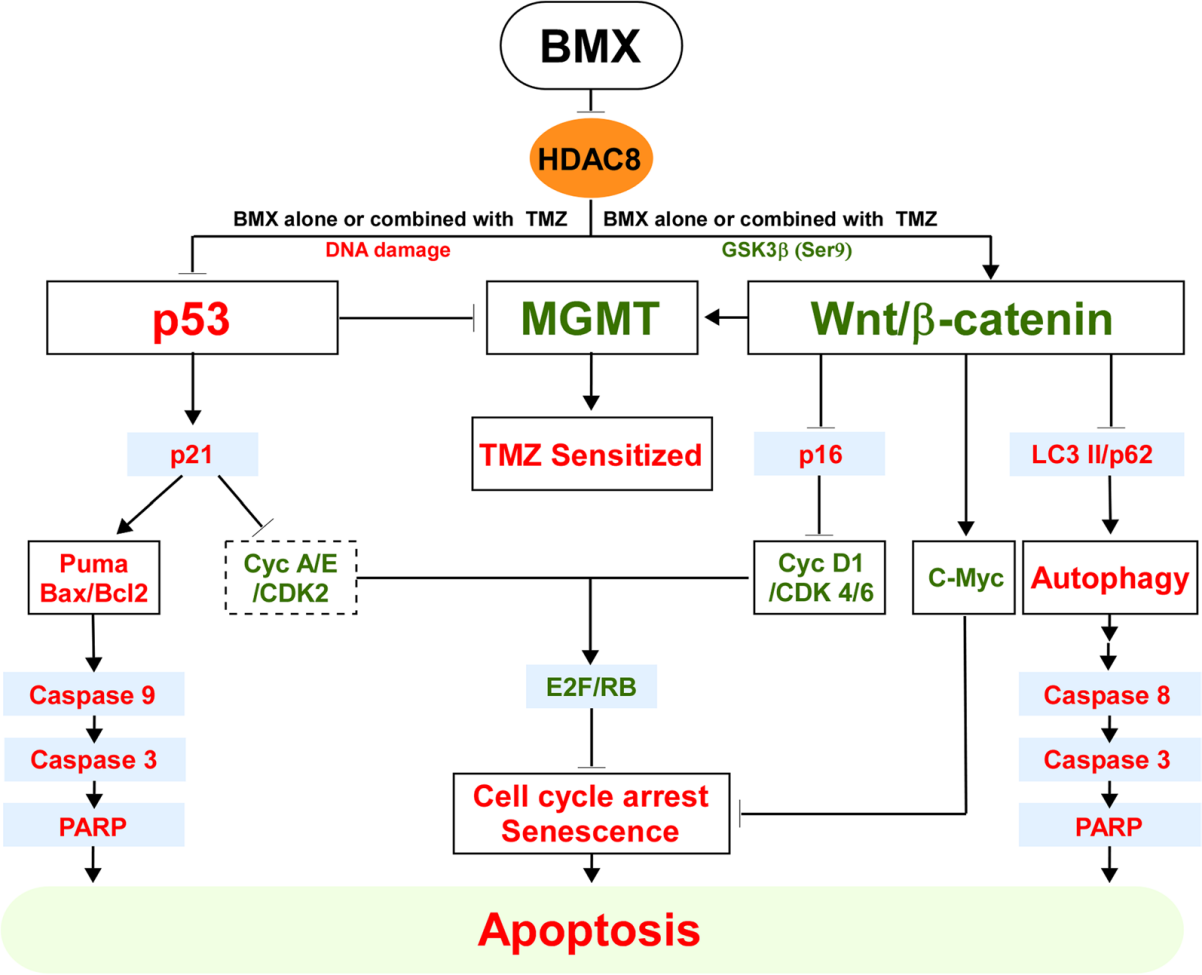
Schematic representation of the proposed communication and signaling pathways. BMX and TMZ combination triggers cell cycle arrest, autophagy, and apoptosis in human colon cancer cells via upregulation of p53/p21/Puma and downregulation of the Wnt/β-catenin/c-Myc/p62 pathways. It is noted that autophagy also contributes to cell death under the opposite status of β-catenin/p62 axis, suggesting that there exists a negative feedback regulation between Wnt/β-catenin and autophagy. Red color indicates upregulation. Green color green indicates downregulation

It has been observed that inhibition of Wnt/β-catenin signaling induces LC3-II and p62 protein expression and is involved in the activation of autophagic flux [9, 50]. Intriguingly, autophagy may also contribute to cell death under the reverse status of the β-catenin/p62 axis, suggesting that there exists negative regulatory feedback between Wnt/β-catenin and autophagy. In our study, NGS results of HT29 and RKO after 6 h of BMX treatment via GSEA showed an enrichment of both upregulated apoptotic and autophagy markers, with NES of 1.65 and 1.96, respectively (Figure S9B). In addition, inactivation of cleaved caspase 3 using specific inhibitors (VAD) did not affect the expression of LC3-II and p62 in the BMX plus TMZ combination-treated cells. However, the expression of cleaved caspase 3 induced by the BMX plus TMZ combination was slightly reversed by treatment with BAF. Moreover, BMX plus TMZ-induced apoptosis and cell death in BAF-treated system suggests that the BMX plus TMZ combination induced cell death through autophagy, and blocking autophagy induced by the BMX plus TMZ combination reduced cell death (Figs. 4 and 6) [47–49]. Additionally, knockdown of p62 might enhance caspase 3-dependent apoptosis and promote cell death, possibly because the decreased level of p62 prevents the degradation of LC3, enabling cell death to occur by autophagy [51]. Thus, these results suggest complex relationships exist among the mechanisms of autophagy, apoptosis, and the Wnt/β-catenin pathway in BMX plus TMZ combination-treated CRC cells (Fig. 8). Of note, in our model HDAC8 did not show direct inhibition of the protein level of MGMT, but we found the decrease in HDAC8 levels conferred downregulation of mRNA expression in MGMT. Significant downregulation of mRNA expression in MGMT was observed in HT29, HCT116, and RKO cells treated with BMX plus TMZ combination or SAHA plus TMZ combination. Therefore, these additional findings suggest either mutated or wild type p53 were negatively correlated with MGMT mRNA expression in these three CRC cell lines via the Wnt/β-catenin pathway (Additional file 1: Figure S12).

Currently, many preclinical studies on non-selective HDACis have been conducted, either alone or in combination with other drugs or treatments [52] Some studies have pointed out that HDAC8-selective inhibitor PCI-34051 can increase p21 expression caused by cell cycle arrest in the G2/M phase. However, these effects were not accompanied by acetylation of histone H3 and histone H4 [53]. Our NGS results in both HT29 and RKO cells showed that cell cycle and transcriptional regulation by p53 were strongly affected by BMX, based on searches of the ConsensusPathDB for 29 commonly downregulated and 71 upregulated genes (Additional file 1: Figure S9C). We also found that regardless of the use of BMX or SAHA combined with TMZ, a significant decrease in the expression level of HDAC8 was observed when apoptosis or autophagy was induced by acetylation of histone H3 and histone H4. Conversely, combined PCI-34051 plus TMZ showed a constant level of HDAC8 protein expression and did not affect cell apoptosis or autophagy, indicating PCI-34051 operates via interaction with the proteasome receptor ADRM1, but may not display a specific HDAC8i [33, 54]. Thus, the present results suggest the possibility that autophagy is a key mechanism by which the HDAC8 gene preferentially kills cancer cells [8, 54, 55]. More interestingly, the synergistic effect of BMX and TMZ showed a stronger cytotoxic and inhibitory effect on colonies in wild-type p53 cell lines, whereas the combination of PCI-34051 and TMZ did not induce this response (Fig. 7). As mentioned above, the degree of MGMT expressed by the three cell lines was decreased to display different sensitivity to the treated drugs. We may also consider the effect of mismatch repair (MMR) and basic excision repair (BER) on drug response and resistance in the three cancer cell lines. Not like direct repair involving MGMT for cancer cell survival, the role of MMR in DNA-induced apoptosis has been explored [12, 16]. The combined TMZ and BMX treatment leading apoptosis (Fig. 2B and C) probably suggests that they induce MMR of the cancer cells. Contrarily, MMR-deficient cancer cells (as manifested by microsatellite instability, MSI) can lead to resistance to anticancer drugs [56]. For example, Meyers et al. [57] reported that HCT116 (MMR deficiency) displayed much more resistant to 5-FU and FdUrd as compared to HCT116 3–6 (MMR proficiency) cells. Additionally, since the three cell lines we used had different genetic backgrounds [12, 16, 58], these cells also responded differently to the combined treatment (Table 1). Drug treatment awakens the repair mechanisms of cancer cells and develops drug resistance. In addition to MGMT and MMR, after all, BER accounts for the majority [12, 16]. Therefore, the mechanism of the effects of the combined treatment proposed in this study only on these three cancer cells needs to be considered the relevance of MMR and BER towards to MGMT and p53 status ([56–58]; Additional file 1: Figure S5) in further explored to determine its utility to better stratify the CRC patients and to select the most beneficial treatments. Eventually, our results might also show a potential role of the combination therapy of BMX plus TMZ by shedding light on their HDAC8-dependent synergistic effect on CRC via autophagic apoptosis, resulting in cell death (Fig. 8). In fact, our findings suggest that BMX is a specific epigenetic eraser inhibitor which can act as a single agent and in combination with reframed TMZ [24].

## Conclusion

These findings demonstrate a remarkable synergic treatment effect capable of eliciting p53-, β-catenin-, and MGMT-dependent apoptotic cell death (Enlarge font of words in Fig. 8) and may also be highly clinically relevant to chemo-regimens for various CRC subtype-based interventions, which can be tailored to a patient’s individual requirements.

## Supplementary Information

**Additional file 1:** Supplementary Figures and Tables.

## Data Availability

All data generated or analysed during this study are included in this published article.

## Abbreviations

CRC: Colorectal cancer
TMZ: Temozolomide
Oxp: Oxaliplatin
CMS1-4: Consensus molecular subtypes
MGMT: O6-methylguanine-DNA-methyltransferase
m7G: 7-Methylguanosine
m3A: 3-Methyladenosine
O6mG: O6-methylguanosine
T: Thymine
PARP-1: Poly ADP-ribose polymerase
BMX: NBM-T-L-BMX-OS01
HDAC8i: Histone deacetylase 8 inhibitor
GBM: Glioblastoma multiforme
ATCC: American type culture collection
FBS: Fetal bovine serum
SA-β-gal: Senescence-associated β-galactosidase
SAHA: Suberoylanilide hydroxamic acid
VPA: Valproic acid
HRP: Horseradish peroxidase
NGS: Next-generation sequencing
DEGs: Differentially expressed gene
GSEA: Gene set enrichment analysis
Dox: Doxorubicin
Z-VAD-FMK: Carbobenzoxy-valyl-alanyl-aspartyl-[O-methyl]-fluoromethylketone
5-FU: Fluoropyrimidine
IRI: Irinotecan
