# Supplementary material for: BMX, a specific HDAC8 inhibitor, with TMZ for advanced CRC therapy: a novel synergic effect to elicit p53-, β-catenin- and MGMT-dependent apoptotic cell death
Source: Cell Commun Signal. 2022 Dec 27;20:200. doi: 10.1186/s12964-022-01007-x (PMC9793577; doi:10.1186/s12964-022-01007-x)
Supplement: Supplementary file 2 — Additional file 1: Supplementary Figures and Tables. [file 12964_2022_1007_MOESM2_ESM.pdf]

Supplemental Information

BMX, a specific HDAC8 inhibitor, with TMZ for advanced CRC therapy: a novel synergic effect to elicit p53-, β-catenin- and MGMT-dependent apoptotic cell death

Huey-Jiun Ko, Shean-Jaw Chiou, Cheng-Yu Tsai, Joon-Khim Loh, Xin-Yi Lin, Thu-Ha Tran, Chia-Chung Hou, Tai-Shan Cheng, Jin-Mei Lai, Peter Mu-Hsin Chang, Feng-Sheng Wang, Chun-Li Su, Chi-Ying F. Huang and Yi-Ren Hong

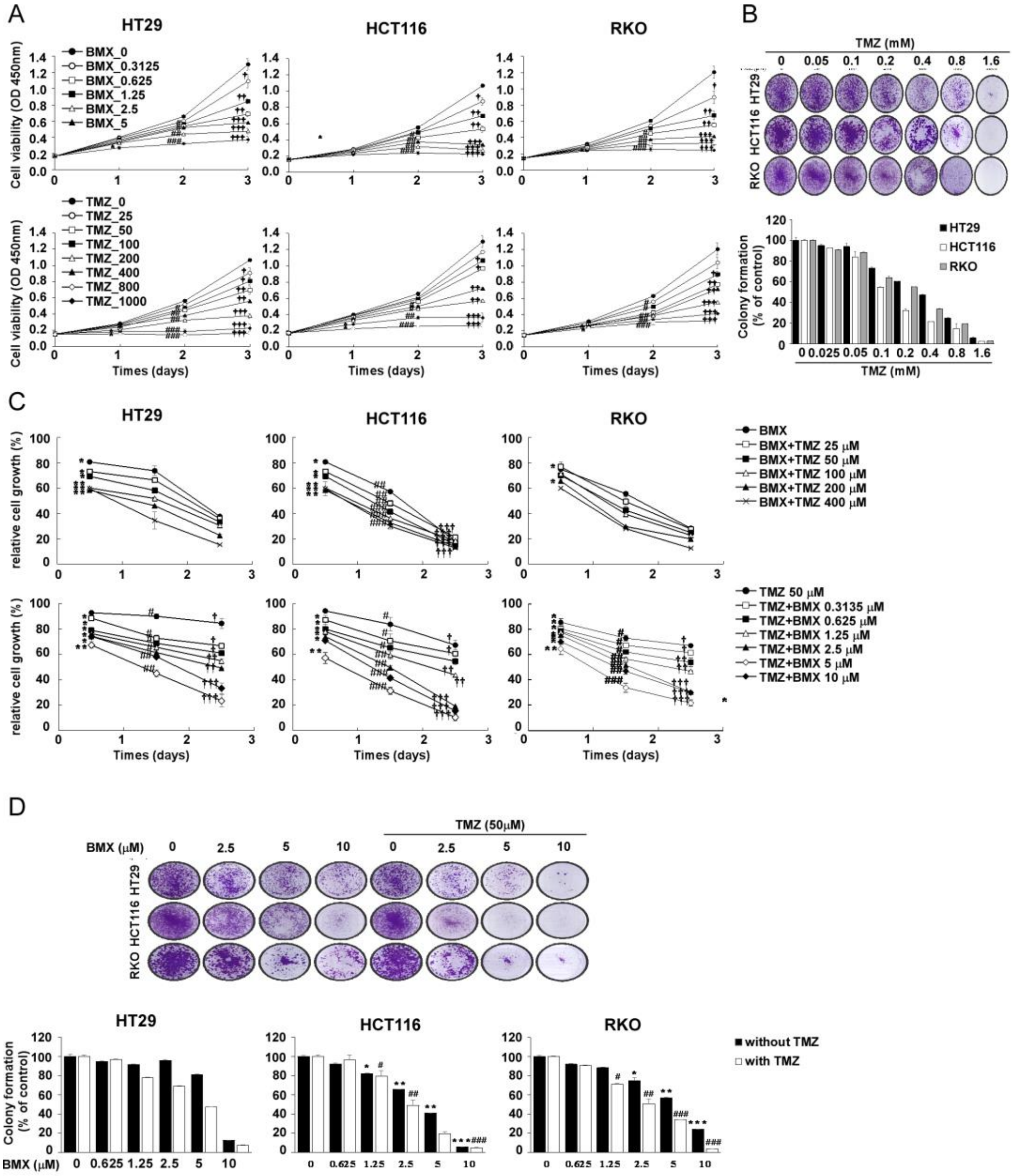

Figure S1. BMX and BMX plus TMZ inhibit the growth and proliferation of HT29, HCT116, and RKO cells. (A) Cell viability of HT29, HCT116, and RKO cells after treating with indicated concentrations of BMX (0.5, 10, 15, 30, or 50 μM) or TMZ (0, 25, 50, 100, 200, 400, 800, 1000 μM) for 1, 2, and 3 days. (B) TMZ repressed colony formation. Cells were treated with different concentrations of TMZ for 48 h. When the colonies formed two weeks later, colony formation assay was carried out. (C) Cell proliferation of HT29, HCT116, and RKO cells after treating with different concentrations of TMZ plus 10 μM BMX, or different concentrations of BMX plus 50 μM TMZ for 1, 2, and 3 days. (D) BMX in combination with TMZ repressed colony formation. Cells were treated with BMX alone (5 and 10 μM) or in combination with 50 μM TMZ. When the colonies formed two weeks later, colony formation assay was carried out.

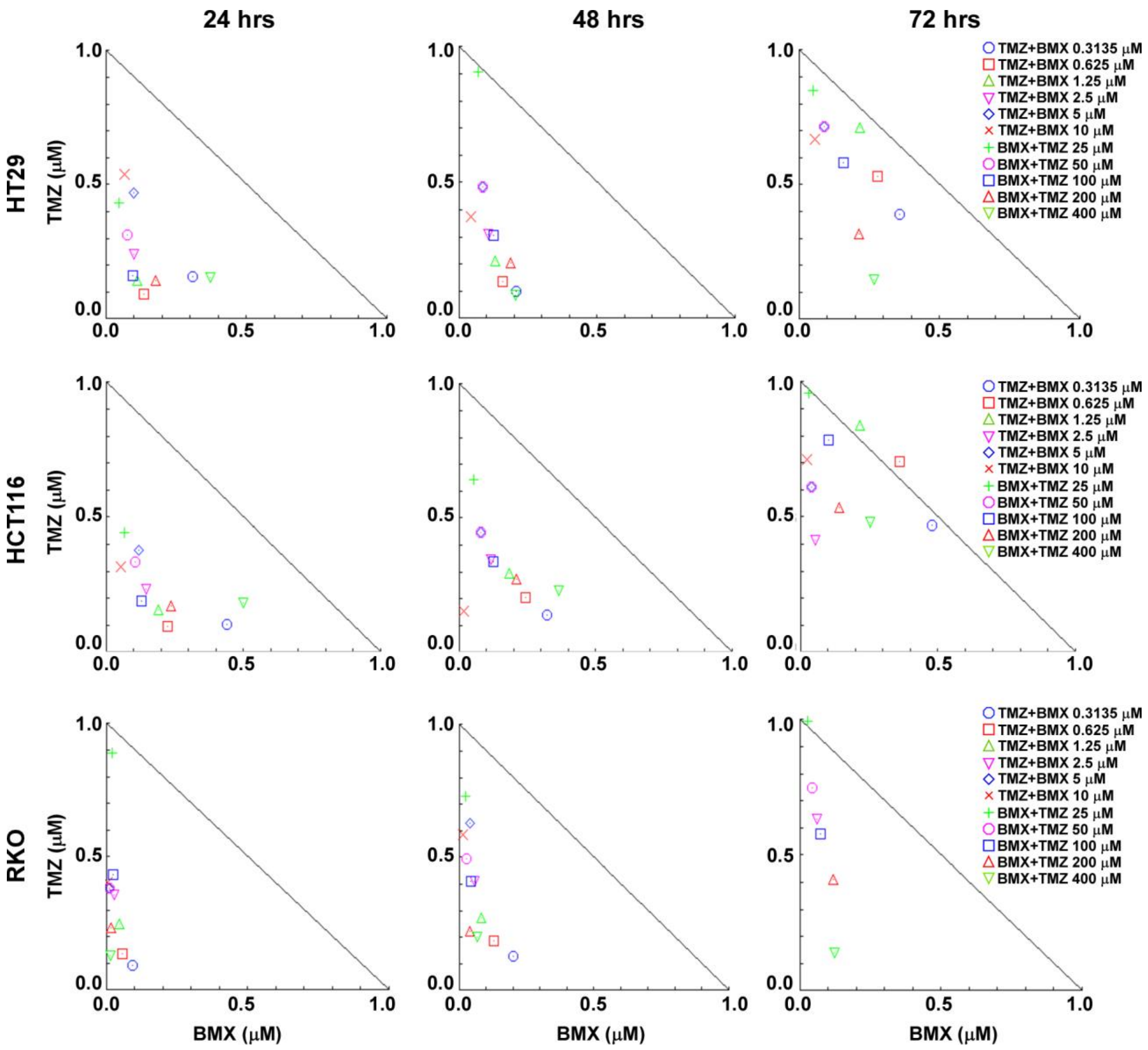

Figure S2. BMX exhibits synergistic effect in combination with TMZ in CRC cells. Drug toxicity was determined via CCK-8 assay for 24, 48, and 72 h in HCT116, HT29, and RKO cells. The drug combination effect was calculated via Compusyn software and demonstrated via the normalized isobologram with each dosage combination presented by one point. Within triangle: synergistic effect; diagonal: additive effect; outside triangle: antagonist effect.

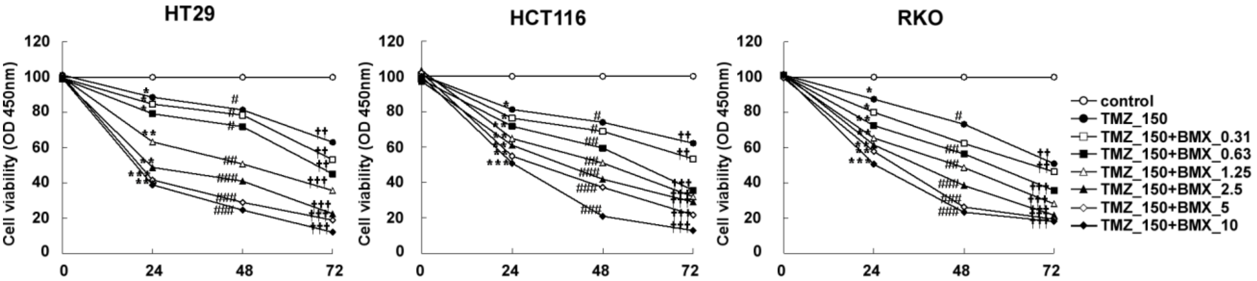

TMZ 150 μM combined with different concentrations of BMX				
TMZ (150 μM)				
	IC50 of BMX (μM)			
	0	24	48	72
HT29	--	2.3±0.1	1.7±0.2	0.4±0.1
HCT116	--	>10	1.5±0.1	0.3±0.1
RKO	--	>10	1.1±0.2	0.1±0.0

Figure S3. BMX plus TMZ combination inhibited cell proliferation in CRC cells. With increased TMZ dose (150 μM), BMX could be lowered to 1-2 μM instead of 5-10 μM. Cell proliferation of HT29, HCT116, and RKO cells after treating with different concentrations of BMX combined with 150 μM TMZ for 1, 2, and 3 days. Specific IC_{50} values for different concentrations of BMX plus 150 μM TMZ in all conditions are displayed in the table. All results are shown as mean ± s.d. from three independent experiments. *$p < 0.05$, **$p < 0.01$, ***$p < 0.001$ vs. control (HT29 cells); #$p < 0.05$, ##$p < 0.01$, ###$p < 0.001$ vs. control (HCT116 cells); †$p < 0.05$, ††$p < 0.01$, †††$p < 0.001$ vs. control (RKO cells).

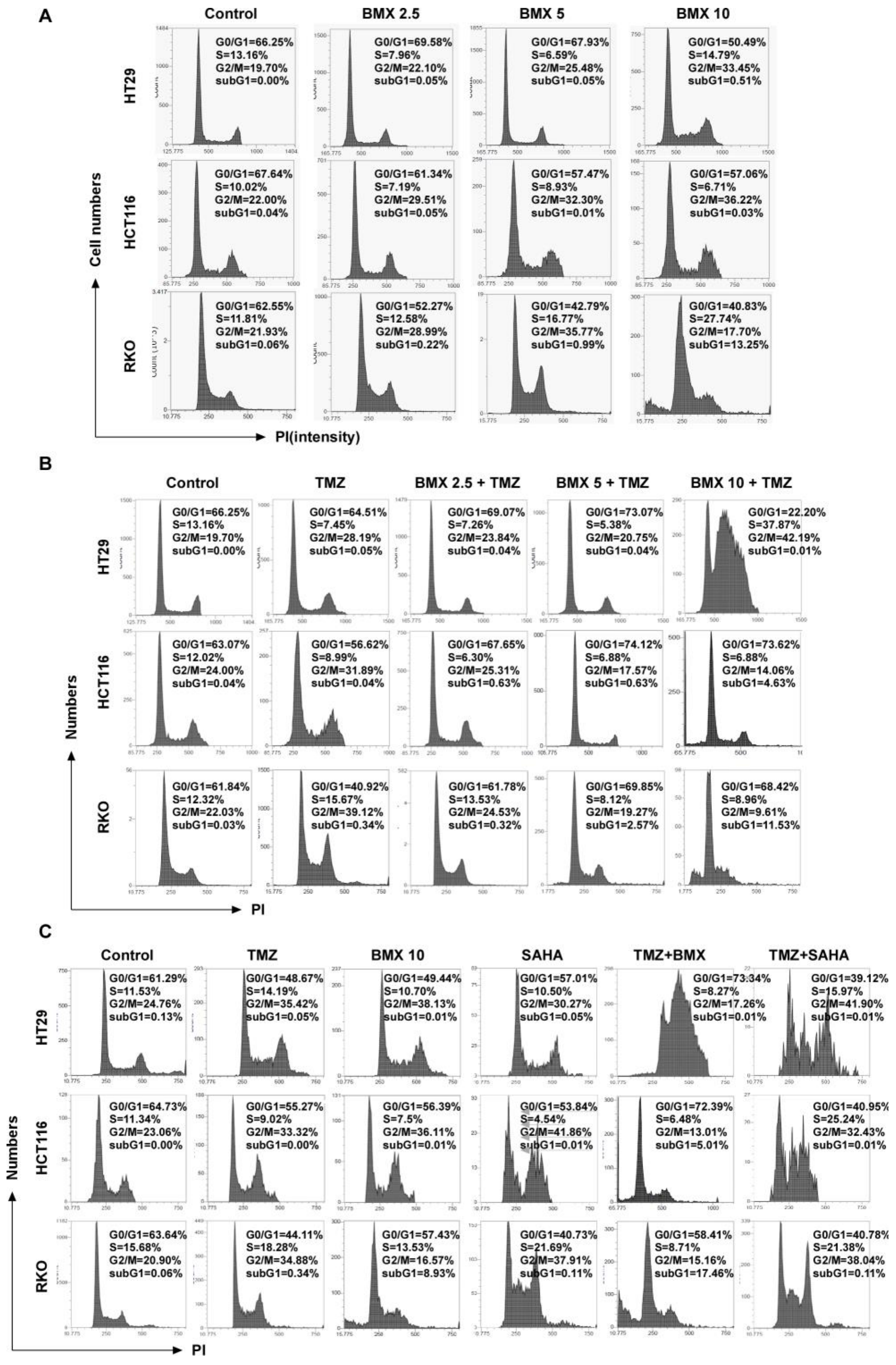

Figure S4. Cell cycle analysis of combination sensitive and resistant CRC cell lines. Cell cycle analysis after 48 hrs treatment with different concentrations of (A) BMX alone or (B) BMX combined with TMZ in HT29, HCT116, and RKO cells and the proportion of cells in each cell cycle phase. (C) Cell cycle analysis after 48 hrs treatment with BMX and SAHA with or without TMZ in HT29, HCT116, and RKO cells, and the proportion of cells in each cell cycle phase. SubG1, cell with polyploid chromosome; >4N, polyploid cell.

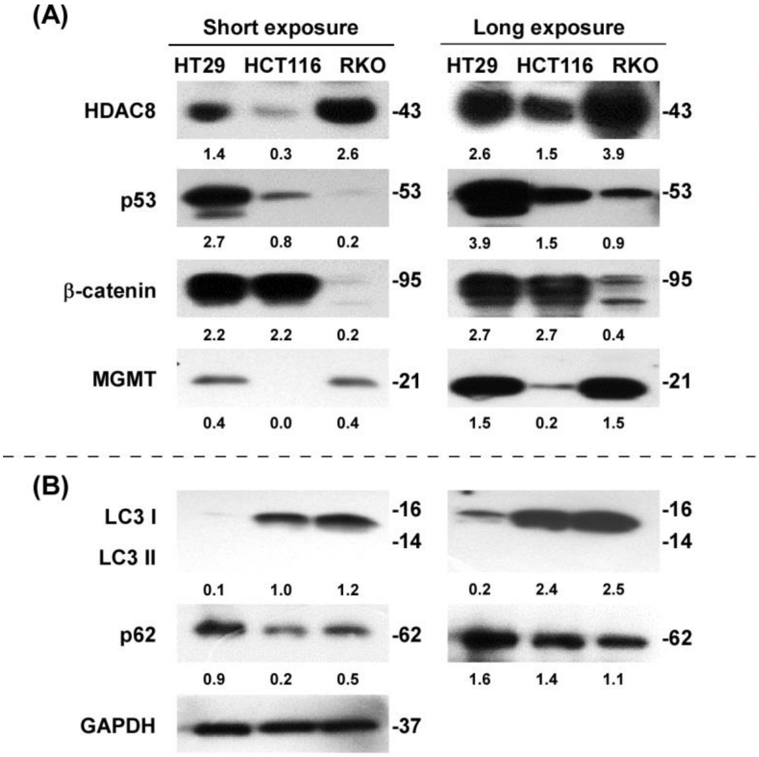

	APC	CTNNB1	TP53
HT29	p.E1554fs	WT	p.R273H
HCT116	WT	p.S45del	WT
RKO	WT	WT	WT

Figure S5. HDAC8, β-catenin, p53, MGMT, LC3 I/II, and p62 expression status of HT29, HCT116, and RKO cells. This figure shows the basic protein expression status of our markers of interest in HT29, HCT116, and RKO cells. (A) The expression pattern of HDAC8, β-catenin, p53, and MGMT on HT29, HCT116, and RKO cells. (B) The expression pattern of LC3 I/II and p62 on HT29, HCT116 and RKO cells. 'Short' and 'Long' Exposure indicate the time of exposure of the immunoblot image. GAPDH was used as the loading control.

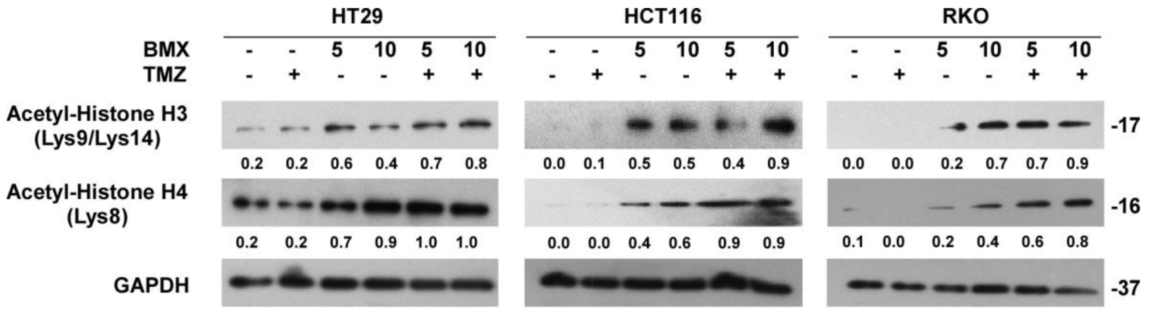

Figure S6. Effects of BMX and BMX plus TMZ combination on the histone acetylation expression in HT29, HCT116, and RKO cells. Cells were treated with different concentrations of BMX with or without TMZ for indicated times. Then, cells were harvested for detection of acetyl-histone H3 (Acetyl Lys9/Lys14) and acetyl-histone H4 (Acetyl Lys8). GAPDH was used as the loading control.

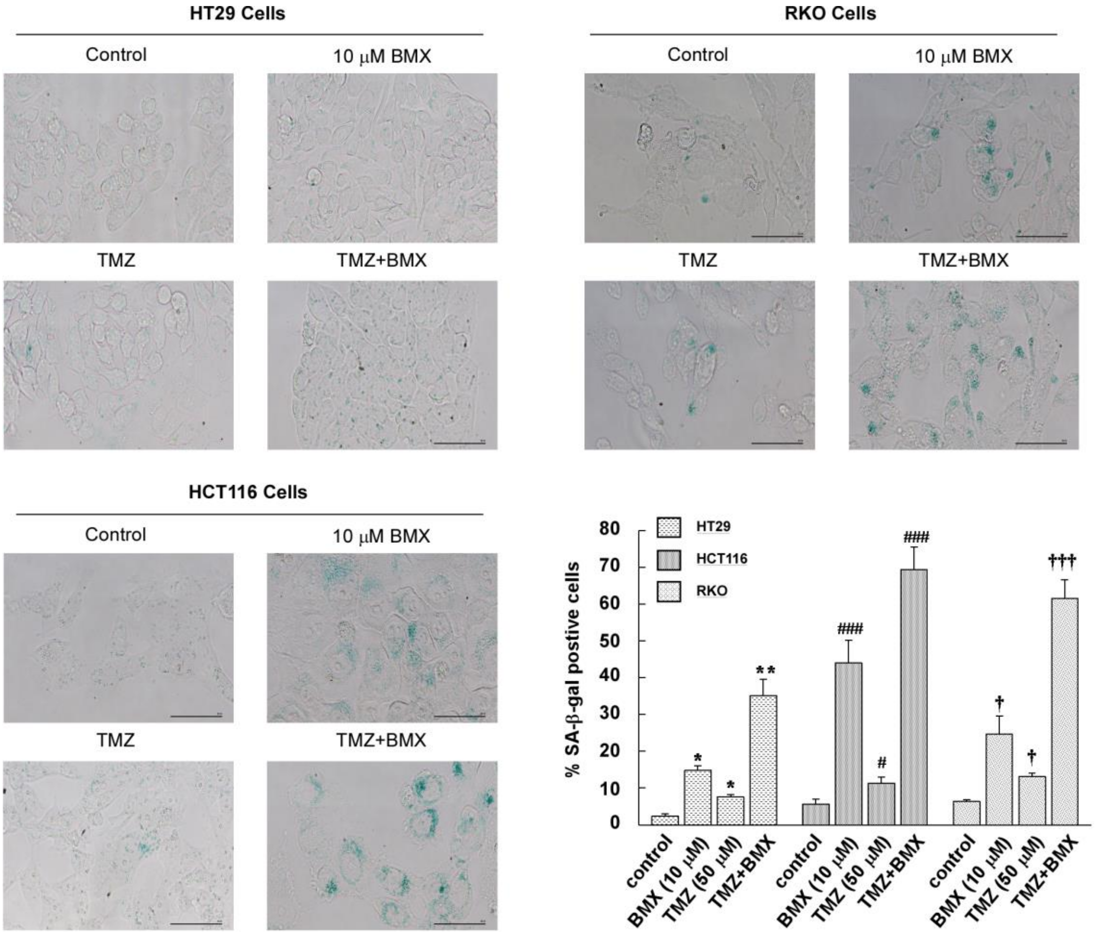

Figure S7. BMX and BMX plus TMZ combination induced cellular senescence in HT29, HCT116, and RKO cells. Senescence-associated β-galactosidase (SAβ-gal) staining of BMX and TMZ combination. Cells were treated with 10 μM of BMX plus TMZ (50 μM) for 48 hrs, and the cells were stained with SAβ-gal (blue cytoplasmic stain). Scale bar, 50 μm. Quantification of SAβ-gal activity. All results are shown as mean ± s.d. from three independent experiments. *$p < 0.05$, **$p < 0.01$, ***$p < 0.001$ vs. control (HT29 cells); #$p < 0.05$, ##$p < 0.01$, ###$p < 0.001$ vs. control (HCT116 cells); †$p < 0.05$, ††$p < 0.01$, †††$p < 0.001$ vs. control (RKO cells).

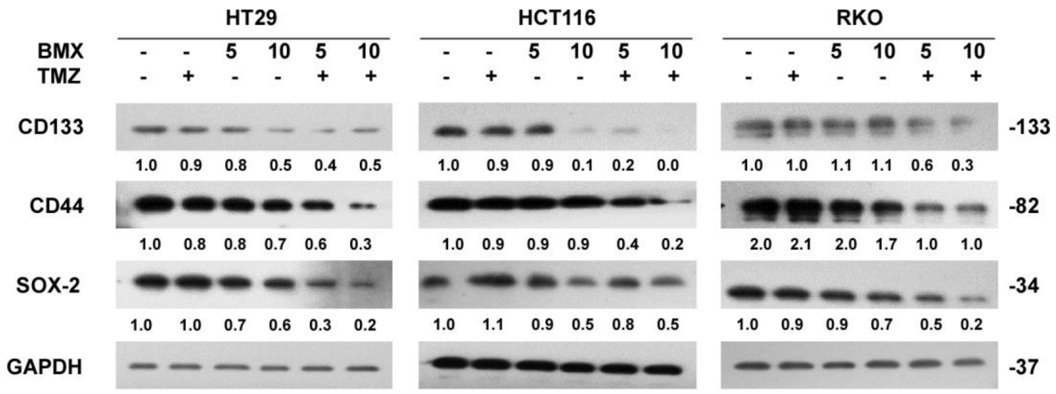

Figure S8. BMX plus TMZ combination reduced CSC formation in HT29, HCT116, and RKO cells. Changes in CD133, CD44, and SOX2 protein levels after receiving 5 and 10 μM BMX with or without 50 μM TMZ for 48 hrs in HT29, HCT116, and RKO cells. GAPDH was used as the loading control.

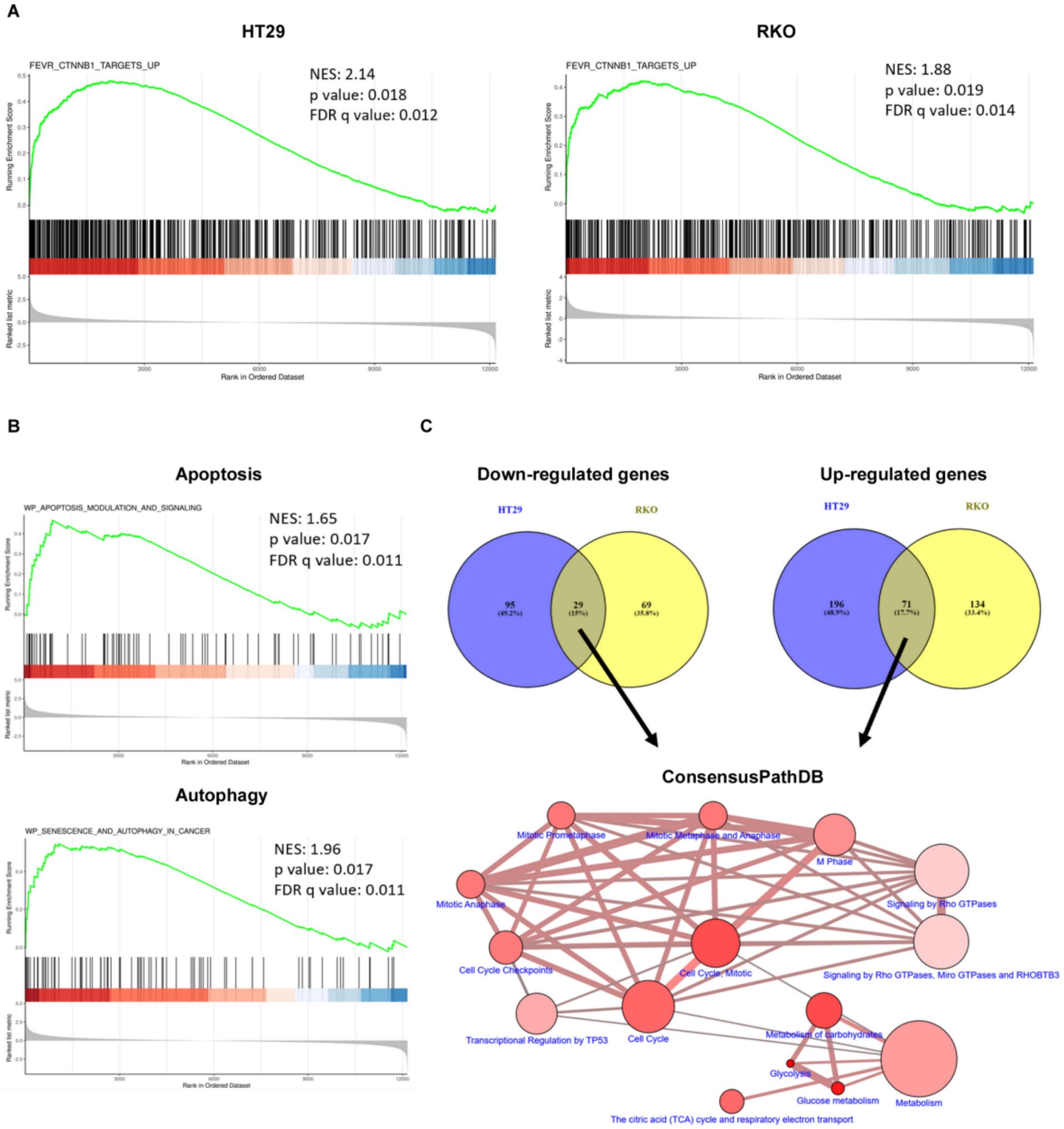

Figure S9. NGS data suggest that BMX could induce apoptosis, autophagy, and cell cycle arrest, and affect Wnt/ β-catenin pathway in CRC cells. (A) BMX affects the Wnt/β-catenin pathway. GSEA was performed to compare the gene set consisting of upregulated genes in intestinal crypt cells upon deletion of *CTNNB1* (56). The analysis was performed using the C2 gene set collections from the MSigDB v.7.2. NES: normalized enrichment score, FDR: false discovery rate. (B) GSEA results of HT29 treated with BMX showed a significant increase in the gene sets involving apoptosis (WP1772) and autophagy (WP615). The analysis was performed using the C2-Wikipathway gene set collections from the MSigDB v.7.2. NES: normalized enrichment score, FDR: false discovery rate. (C) Mutually altered gene expressions in HT29 and RKO cells after BMX treatment were analyzed via ConsensusPathDP to determine whether BMX might affect cell cycle and transcriptional regulation by p53.

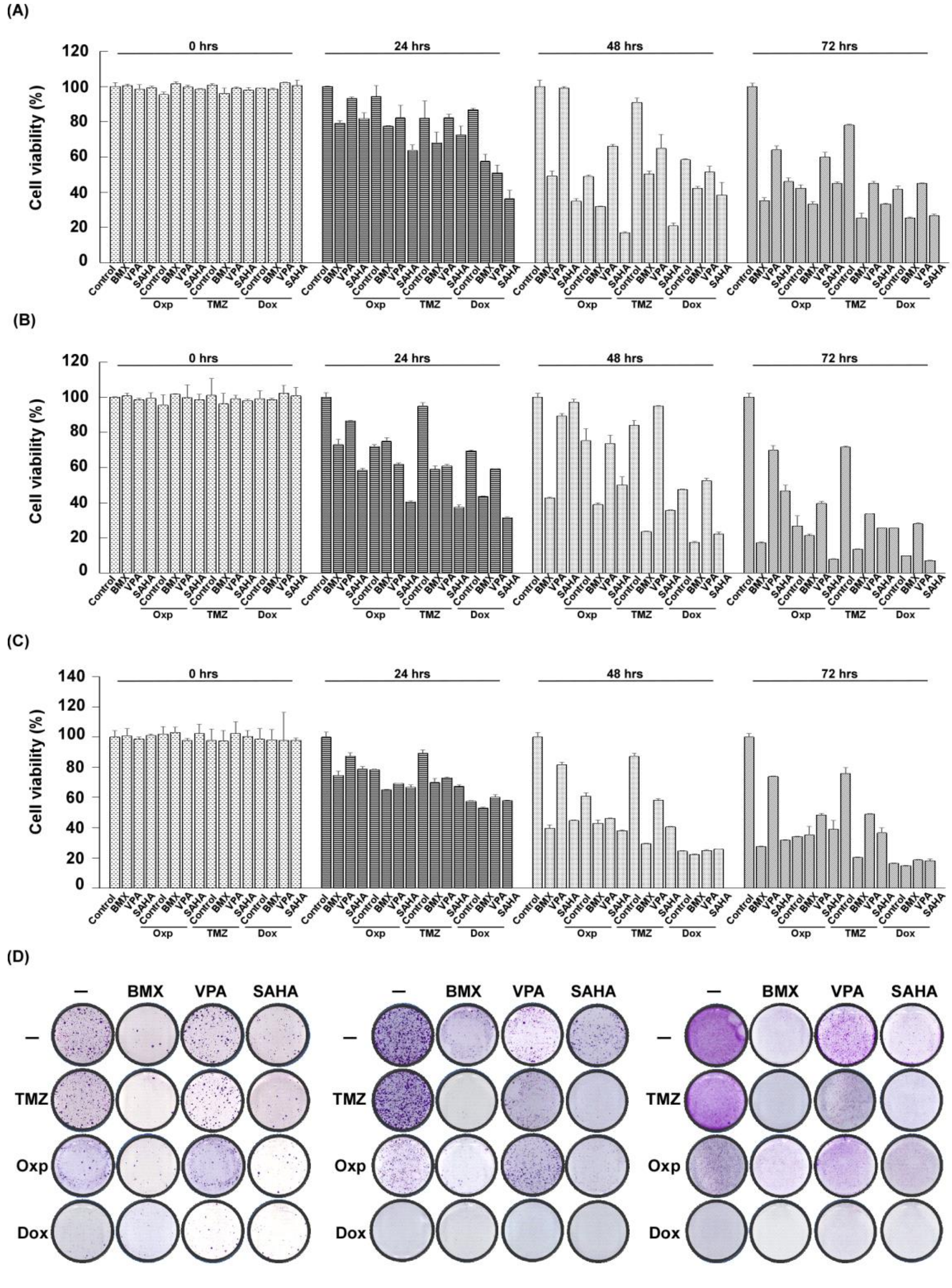

Figure S10. BMX, VPA, SAHA, TMZ, Oxp, and Dox combination inhibited cell proliferation in CRC cells. The proliferation of BMX, VPA, SAHA with or without TMZ, Oxp, and Dox for 0, 24, 48, and 72 hrs in (A) HT29, (B) HCT116, and (C) RKO cells with treatment durations were assayed using the CCK-8 method. (D) Colony formation capability assay with different treatments of BMX, VPA, SAHA with or without TMZ, Oxp, and Dox in HT29, HCT116, and RKO cells.

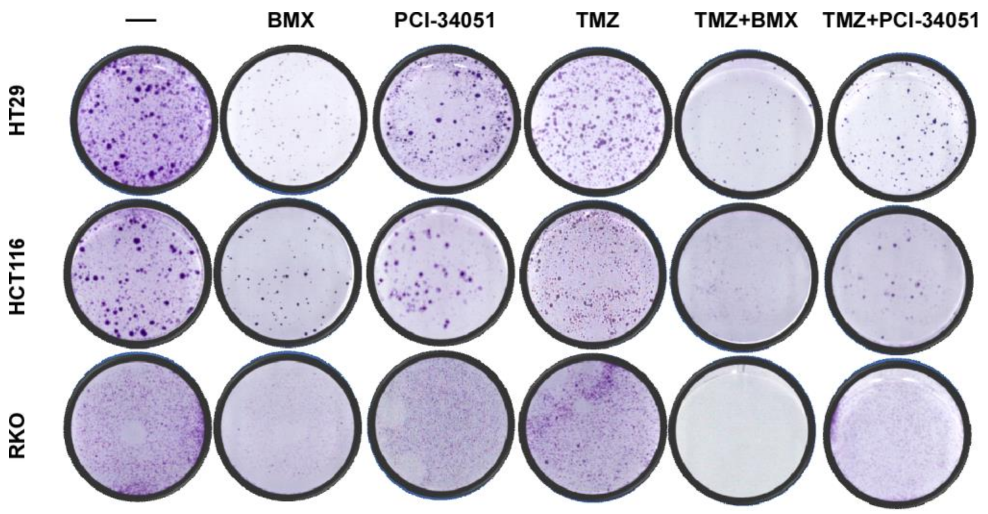

Figure S11. BMX plus PCI-34051 combination inhibited colony formation capability in CRC cells. Colony formation capability assay with different treatments of BMX and PCI-34051 with or without 50 μM TMZ in HT29, HCT116, and RKO cells.

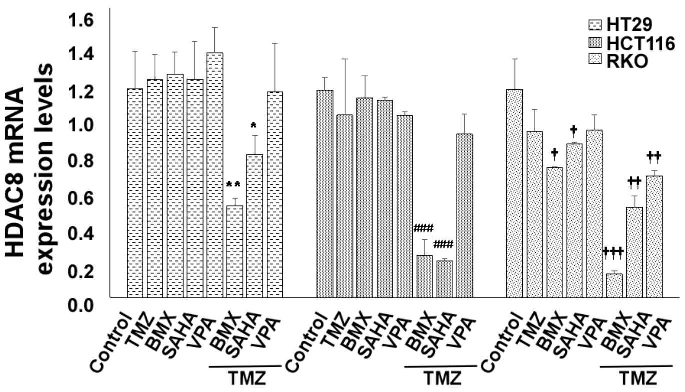

Figure S12. MGMT expression status of HT29, HCT116, and RKO cells. MGMT mRNA expression levels stimulated with BMX (5 μM), VPA (4 mM), SAHA (2 μM) with or without TMZ were determined using qRT-PCR assays.

Supplementary Table 1. Key resources table.

Reagent Type (Species) or Resource	Designation	Source or Reference	Identifiers	Additional Information
Antibody	Rabbit anti-Acetyl-Histone H3(Lys9/Lys14)	Cell signaling (Beverly, MA, USA)	s9677	1:1000
Antibody	Rabbit anti-Acetyl-Histone H4(Lys8)	Cell signaling (Beverly, MA, USA)	s2594	1:1000
Antibody	Mouse anti-GAPDH	Santa Cruz (Santa Cruz, CA, USA)	sc-32233	1:1000
Antibody	Mouse anti-p53	Santa Cruz (Santa Cruz, CA, USA)	sc-126	1:1000
Antibody	Rabbit anti-Acetyl-p53(Lys382)	Cell signaling (Beverly, MA, USA)	s2525	1:1000
Antibody	Rabbit anti- Phospho-p53 (Ser15)	Cell signaling (Beverly, MA, USA)	9284	1:1000
Antibody	Rabbit anti-p21	Cell signaling (Beverly, MA, USA)	s2947	1:1000
Antibody	Mouse anti-p16	Santa Cruz (Santa Cruz, CA, USA)	sc-56330	1:1000
Antibody	Rabbit anti-MGMT	Cell signaling (Beverly, MA, USA)	s2739	1:1000
Antibody	Rabbit anti- phosphor-γ-H2AX (S139)	Elabscience	E-AB-68087	1:1000
Antibody	Mouse anti-E2F1	Santa Cruz (Santa Cruz, CA, USA)	SC-251	1:1000
Antibody	Mouse anti-E2F3	Santa Cruz (Santa Cruz, CA, USA)	SC-56665	1:1000
Antibody	Rabbit anti-Cleaved Caspase 3	Cell signaling (Beverly, MA, USA)	s9661	1:1000
Antibody	Rabbit anti-Cleaved Caspase 9	Cell signaling (Beverly, MA, USA)	s9501	1:1000
Antibody	Rabbit anti-Cleaved Caspase 7	Cell signaling (Beverly, MA, USA)	s9491	1:1000
Antibody	Rabbit anti-Cleaved Caspase 8	Cell signaling (Beverly, MA, USA)	s9496	1:1000
Antibody	Rabbit anti-PARP	Cell signaling (Beverly, MA, USA)	s9542	1:1000
Antibody	Rabbit anti-Bax	Cell signaling (Beverly, MA, USA)	s2772	1:1000
Antibody	Rabbit anti-Bcl-2	Cell signaling (Beverly, MA, USA)	s2870	1:1000
Antibody	Rabbit anti-Bid	Cell signaling (Beverly, MA, USA)	s2002	1:1000
Antibody	Rabbit anti-Bim	Cell signaling (Beverly, MA, USA)	s2819	1:1000
Antibody	Rabbit anti-Bak	Cell signaling (Beverly, MA, USA)	s3814	1:1000
Antibody	Rabbit anti-Puma	Cell signaling (Beverly, MA, USA)	s4976	1:1000
Antibody	Rabbit anti- β-catenin	Cell signaling (Beverly, MA, USA)	s9562	1:1000

Antibody	Rabbit anti-Phospho-β-catenin (Ser/33/37/41)	Cell signaling (Beverly, MA, USA)	s9561	1:1000
Antibody	Rabbit anti-Phospho-GSK3β (Ser 9)	Cell signaling (Beverly, MA, USA)	s9323s	1:1000
Antibody	Mouse anti-GSK3β	BD Biosciences	610202	1:1000
Antibody	Rabbit anti-c-Myc	Abcam (Cambridge, MA, USA)	ab32072	1:1000
Antibody	Mouse anti-Cyclin D1	Santa Cruz (Santa Cruz, CA, USA)	sc-8396	1:1000
Antibody	Mouse anti-α-tubulin	Sigma-Aldrich (St. Louis, MO, USA)	T5168	1:5000
Antibody	Mouse anti-p62	Abcam (Cambridge, MA, USA)	ab56416	1:2000
Antibody	Rabbit anti-LC3I/II	Cell signaling (Beverly, MA, USA)	s3868	1:1000
Antibody	Rabbit anti-CD133	Cell signaling (Beverly, MA, USA)	s64326	1:1000
Antibody	Rabbit anti-CD44	Proteintech (, USA)	15675-1-AP	1:2000
Antibody	Rabbit anti-SOX2	Abcam (Cambridge, MA, USA)	ab97959	1:1000
Antibody	Rabbit anti-HDAC8	ABclonal	a8865	1:1000
